# Procedural interventions for chronic migraine: an umbrella review intervention-specific evidence map

**DOI:** 10.3389/fneur.2026.1915292

**Published:** 2026-10-12

**Authors:** Lijie Xia, Dianhui Yang, Yan Lu, Yunfei Gong, Chengtao Zhang

**Affiliations:** 1 College of Acupuncture-Moxibustion and Tuina, Shandong University of Traditional Chinese Medicine, Jinan, Shandong, China; 2 Department of Acupuncture and Moxibustion, Affiliated Hospital of Shandong University of Traditional Chinese Medicine, Jinan, Shandong, China; 3 Shandong Provincial Research Laboratory for the Efficacy of Traditional Chinese Medicine, Jinan, Shandong, China

**Keywords:** chronic migraine, evidence mapping, nerve block, procedural interventions, umbrella review

## Abstract

**Objective:**

To synthesize and map intervention-specific evidence on the efficacy, clinical relevance, and safety of procedural interventions for chronic migraine (CM).

**Methods:**

We conducted a PROSPERO-registered umbrella review in accordance with PRISMA 2020 and methodological guidance for umbrella reviews. Eligible systematic reviews evaluated onabotulinumtoxinA, greater occipital nerve block (GONB), other peripheral nerve blocks and percutaneous procedures, acupuncture, implanted occipital nerve stimulation (ONS), or surgical interventions. Evidence concerning the addition of CGRP-targeted monoclonal antibody therapy to ongoing onabotulinumtoxinA treatment was retained separately as contextual evidence on adjunctive pharmacotherapy and was not classified as a procedural intervention. Methodological quality was assessed using A MeaSurement Tool to Assess systematic Reviews 2 (AMSTAR 2), certainty of evidence using Grading of Recommendations Assessment, Development and Evaluation (GRADE), and primary-study overlap using the corrected covered area (CCA). Evidence was synthesized separately by intervention, outcome, comparator, and follow-up period. Pooled estimates from different systematic reviews were not statistically combined, and clinically heterogeneous evidence was summarized narratively.

**Results:**

Seventeen reviews were included, of which 12 contained at least one meta-analysis. The strength and maturity of evidence varied substantially across interventions. OnabotulinumtoxinA had the most established evidence base and the clearest role in CM prevention. For GONB, low-certainty evidence from clinically comparable randomized trials suggested a possible short-term reduction in headache day and headache severity, although sustained benefit remains uncertain. Evidence for combined onabotulinumtoxinA–CGRP monoclonal antibody therapy was derived predominantly from uncontrolled observational cohorts and should therefore be considered preliminary. Implanted ONS showed possible benefit in selected patients with refractory CM, but evidence was limited by small study numbers, imprecision, heterogeneous outcomes, and device- or procedure-related risks. Evidence for acupuncture, other peripheral nerve blocks, percutaneous procedures, and surgical interventions was limited by clinical heterogeneity, indirectness, uncontrolled study designs, or uncertain applicability specifically to CM. TRAEs profiles differed substantially among intervention categories and were therefore not pooled across procedures. Most intervention–outcome evidence was rated as low or very low certainty.

**Conclusions:**

Procedural interventions for CM should not be interpreted as a homogeneous therapeutic class. OnabotulinumtoxinA is supported by the most mature evidence and established clinical positioning, whereas GONB may provide a short-term, protocol-specific benefit with low certainty. Evidence for other procedures remains preliminary, heterogeneous, or restricted to selected patient populations. Clinical interpretation and treatment selection should therefore be intervention-specific and patient-specific. Future research should prioritize standardized diagnostic criteria, treatment protocols, comparators, clinically meaningful outcomes, TRAEs reporting, and longer-term follow-up. **Systematic review registration:** https://www.crd.york.ac.uk/PROSPERO/view/CRD420261415558, identifier: CRD420261415558.

## 1 Introduction

CM is a highly disabling neurological disorder characterized by headache occurring on at least 15 days per month for more than 3 months, with migraine features present on at least 8 days per month (1). Attacks are frequently accompanied by nausea, vomiting, photophobia, and phonophobia (2). Migraine is among the leading causes of neurological disability worldwide and imposes a substantial medical, social, and economic burden (3). Although migraine affects approximately 14%−15% of the global population, CM represents a smaller but clinically important subgroup, with an estimated prevalence of approximately 2% and a disproportionate impact on health-related quality of life, healthcare utilization, work productivity, and daily functioning (4, 5).

Contemporary management of CM encompasses oral preventive medications, CGRP pathway–targeted therapies, onabotulinumtoxinA, behavioral interventions, non-invasive neuromodulation, and a range of clinician-delivered procedural interventions (6, 7). The therapeutic positioning of these options has evolved considerably in the CGRP era. Current clinical recommendations increasingly emphasize individualized treatment selection based on previous treatment response, comorbidities, tolerability, contraindications, accessibility, patient preferences, and the quality and certainty of intervention-specific evidence (8–10). The focus on clinician-delivered procedural interventions was not based on an assumption of superior efficacy or adherence relative to home-based treatments, but on their distinct procedural burden, safety considerations, technical requirements, and patient-selection implications, together with the fragmented and heterogeneous nature of the existing review-level evidence.

For the purposes of this umbrella review, procedural interventions were defined as clinician-delivered treatments involving a prespecified physical or technical procedure, including injection- or needling-based interventions, peripheral nerve blocks, implanted neuromodulation, and surgical procedures. CGRP-targeted monoclonal antibody therapy was considered a pharmacological preventive treatment rather than a procedural intervention. Evidence concerning its addition to ongoing onabotulinumtoxinA treatment was retained only as contextual adjunctive pharmacotherapy. Indeed, these interventions occupy distinct positions within contemporary headache practice. OnabotulinumtoxinA (11) is an established evidence-based preventive treatment for CM. The addition of CGRP-targeted monoclonal antibody therapy to ongoing onabotulinumtoxinA treatment represents an emerging adjunctive pharmacological strategy, mainly evaluated in patients with an incomplete response to single-agent prevention; however, the current evidence remains largely observational (12). Acupuncture (13) represents a traditional Chinese medicine therapy. Nerve block (14) including greater occipital nerve block, are clinic-based procedures using local anesthetic around selected nerves. ONS (15) is an implanted neuromodulation procedure mainly studied in highly selected patients with refractory CM. In contrast, CM surgery (16) remains controversial because the available evidence is heterogeneous, is frequently derived from small or non-randomized studies, and is subject to important methodological limitations. These interventions can be broadly distinguished according to whether their therapeutic effects depend primarily on pharmacological agents. OnabotulinumtoxinA injections and local-anesthetic nerve blocks represent pharmacological procedures, whereas acupuncture, implanted ONS, and CM surgery represent non-pharmacological procedures, although they differ substantially in mechanism and delivery.

Previous systematic reviews and meta-analyses (17) have evaluated individual procedural interventions for CM; however, the evidence remains fragmented across interventions and heterogeneous in patient populations, procedural protocols, comparators, outcomes, and follow-up periods. Such differences may limit the interpretability of pooled estimates and obscure clinically relevant distinctions among procedures. In addition, evidence volume does not necessarily reflect evidence certainty because multiple reviews may rely on overlapping primary studies, while methodologically rigorous reviews may still yield low-certainty conclusions. Accordingly, an umbrella-level synthesis is needed to evaluate not only efficacy signals but also methodological quality, certainty of evidence, primary-study overlap, clinical relevance, and intervention-specific positioning within a common framework. The present review therefore aimed to synthesize and map the efficacy, safety, methodological quality, and certainty of evidence for procedural interventions in CM, with emphasis on clinical interpretability, patient selection, and benefit–risk considerations.

## 2 Materials and methods

### 2.1 Study design

This umbrella review followed PRISMA 2020 and methodological guidance for umbrella reviews. The protocol was registered in PROSPERO (CRD420261415558).

### 2.2 Eligibility criteria

**Population:** Reviews were eligible when they evaluated participants with CM diagnosed according to the International Classification of Headache Disorders, 3rd edition (ICHD-3). Reviews containing mixed migraine populations were eligible only when CM data were separately extractable or constituted a clinically interpretable subgroup.

**Interventions:** Eligible procedural interventions included onabotulinumtoxinA, acupuncture, peripheral nerve blocks including GONB, neuromodulation techniques, and surgical procedures.

Evidence concerning the addition of CGRP-targeted therapy to ongoing onabotulinumtoxinA treatment was retained separately as contextual evidence on adjunctive pharmacotherapy and was not classified as an independent procedural intervention.

**Comparators:** Eligible comparators included sham or placebo procedures, usual care, standard pharmacological treatment, active comparators, or no intervention, as reported by the included reviews.

**Outcomes:** The outcomes of interest were defined *a priori* and included headache days, headache frequency, headache duration, headache severity, Headache Impact Test-6 (HIT-6), Migraine Disability Assessment (MIDAS), Migraine-Specific Quality of Life Questionnaire (MSQ), and treatment-related adverse events (TRAEs). The availability of each outcome for synthesis depended on reporting in the eligible source reviews. Headache days, headache frequency, headache duration, and headache severity were retained as distinct outcomes.

**Study design:** systematic reviews, with or without meta-analysis, that synthesized randomized controlled trials and/or observational studies evaluating procedural interventions for CM.

### 2.3 Information sources and update search

The search strategy was developed in consultation with two information specialists. Embase, PubMed, the Cochrane Library, Web of Science, and CNKI were searched from March 2015 to July 4, 2026. The complete search strategy is provided in Supplementary Table S1.

### 2.4 Selection process

Two reviewers (XLJ and GYF) independently screened titles and abstracts and subsequently assessed the full texts of potentially eligible reviews. Disagreements were resolved through discussion and, when necessary, consultation with a senior reviewer (ZCT). The primary studies included in each eligible review were then identified and assessed against the predefined eligibility criteria. Reviews were excluded when fewer than 20% of their primary studies met these criteria, based on a prespecified threshold intended to ensure sufficient relevance and interpretability of the evidence. Reviews that otherwise met the eligibility criteria were retained for qualitative evidence mapping, even when their published pooled estimates were unsuitable for quantitative synthesis. Meta-analytic estimates were not re-pooled when they combined clinically non-comparable populations, co-interventions, treatment schedules, procedural laterality, outcome definitions, or follow-up intervals.

### 2.5 Data extraction

Pooled estimates and review characteristics were extracted into Microsoft Excel. Two reviewers (XLJ and GYF) performed data extraction, and a random 25% sample of extracted records was cross-checked. Discrepancies were resolved by senior reviewers (YDH). Extracted items included publication, sample size, intervention, comparator, outcome, effect measure [mean difference (MD), standardized mean difference (SMD), relative risk (RR), or odds ratio (OR)], 95% confidence interval (CI), *P* value, and heterogeneity (*I*^2^), follow-up times, AMSTAR 2 and GRADE. Headache day, headache frequency, headache duration, and headache severity were retained as distinct outcomes. Mean differences and standardized mean differences were not combined; odds ratios and risk ratios were also kept separate. Follow-up was organized as short term ( ≤ 4 weeks), medium term (>4–12 weeks), and long term (>12 weeks). Each participant cohort contributed only one estimate to a given intervention-outcome-time-window synthesis. When more than one estimate was available within the same time window, the review-defined primary time point or the time point closest to the end of the window was selected. Additional details are provided in Supplementary Table S2.

### 2.6 Selection of evidence when reviews overlapped

When multiple reviews evaluated the same intervention–outcome–follow-up combination, a single index review was selected for the primary quantitative interpretation using a hierarchical set of criteria: (1) concordance with the predefined PICOS criteria; (2) availability of separately extractable data for CM; (3) clinical comparability of the intervention protocol, comparator, outcome definition, and follow-up interval; (4) methodological quality assessed using AMSTAR 2; (5) appropriate assessment and incorporation of primary-study risk of bias; (6) recency of the literature search; and (7) the number of eligible primary studies. The number of included studies was therefore considered a secondary measure of comprehensiveness rather than the primary selection criterion. Other eligible reviews were retained for qualitative comparison, assessment of consistency, and identification of methodological limitations.

### 2.7 Methodological quality and certainty of evidence

AMSTAR 2 was used to assess review methodology (18). SRMAs were rated as “no,” “partial yes,” or “yes.” Nine non-critical items and seven critical items that influence the validity of the assessment were included. These items cover protocol registration, comprehensiveness of search strategies, reasons for excluding studies, assessment of risk of bias in studies, appropriateness of statistical methods in meta-analyses, use of risk of bias in the interpretation/discussion process, and evaluation of publication bias (19). Although these scoring criteria could be adjusted based on the context of the umbrella review, no changes were made in this study. Our methodology was rigorous, and the credibility of our umbrella review was rated as high (no critical flaws and at most one non-critical flaw), moderate (more than one non-critical flaw), low (one critical flaw, regardless of non-critical flaws), or very low (more than one critical flaw, regardless of non-critical flaws) (20).

GRADE was independently reassessed by the review team at the intervention–outcome level. Randomized controlled trial evidence initially started at high certainty, whereas observational evidence initially started at low certainty. Certainty was downgraded, where appropriate, for risk of bias, inconsistency, indirectness, imprecision, and publication bias, and the final certainty was rated as high, moderate, low, or very low (21).

AMSTAR 2 and GRADE were displayed separately because a methodologically rigorous review may still contain small, biased, or imprecise trials and therefore yield low-certainty evidence.

### 2.8 Clinical relevance

Statistical significance was distinguished from clinical relevance. A reduction of at least 6 points in HIT-6 was considered a meaningful within-person response in CM, while recognizing that this threshold is not directly interchangeable with a between-group mean difference (22). For MIDAS, interpretation considered both a change of approximately 4–4.5 points in patients with lower baseline disability and an approximately 30% reduction in patients with baseline MIDAS >20 (23, 24). For headache-day outcomes, absolute change was interpreted alongside ≥30% or ≥50% responder rates when available, consistent with CM trial guidance (25). These thresholds were used to support clinical interpretation and were not treated as formal criteria for statistical significance.

### 2.9 Statistical synthesis, overlap, and evidence mapping

Quantitative results were synthesized separately according to intervention, outcome, comparator, and follow-up time. When multiple systematic reviews evaluated the same clinical question, the review that best matched the predefined PICOS criteria and the hierarchical selection criteria described in Section 2.6 was selected as the main source of evidence. We did not statistically combine pooled estimates from different systematic reviews. When an eligible source review provided a clinically appropriate meta-analytic estimate, the published pooled estimate was retained and presented directly. Review-level pooled estimates were prioritized for quantitative presentation. Individual primary-study findings were reported only when they provided clinically relevant contextual information, helped explain heterogeneity, or addressed outcomes not adequately represented by pooled estimates; such findings were not treated as independent quantitative evidence. Estimates were not used when the underlying studies differed substantially in patient population, intervention protocol, comparator, outcome definition, or follow-up period. In such cases, findings were summarized narratively. To avoid double counting, overlapping primary studies and participant cohorts were identified before quantitative interpretation. Estimates from overlapping reviews were not treated as independent evidence. MDs were not combined with SMDs, and RRs were not combined with ORs. Controlled comparisons were also presented separately from uncontrolled changes from baseline. No overall pooled effect was calculated across different procedural intervention categories. Treatment-related adverse events were also summarized separately for each intervention because their nature, severity, and clinical implications differed substantially across procedures. Primary-study overlap was assessed using the CCA and citation matrices. The evidence map summarized intervention category, evidence volume, direction of benefit, GRADE certainty, AMSTAR 2 methodological quality, safety considerations, and current clinical position. Figures and evidence visualizations were generated using R version 4.6.0 (R Foundation for Statistical Computing, Vienna, Austria).

## 3 Results

The database search identified 442 records, of which 154 duplicates were removed. After title and abstract screening, 202 records were excluded. Of the remaining 86 records, 9 full texts were unavailable and 77 were assessed for eligibility. After exclusion of 60 records (reasons are detailed in Supplementary Table S3), 17 (26–42) reviews were included in the umbrella review. 12 reviews contained meta-analyses, and 5 were systematic reviews without meta-analysis.

### 3.1 Study selection and review characteristics

The included reviews were published between 2015 and 2026, and sample sizes ranged from 32 to 3,565 participants. Of the 17 reviews, 1 (26) evaluated Procedural interventions, 1 (27) acupuncture, 6 (28–33) onabotulinumtoxinA, 1 (34) onabotulinumtoxinA and CGRP-targeted therapy, 3 (35, 39, 40) ONS, 4 (36–38, 41) nerve block, and 1 (42) surgical intervention. The study-selection process is summarized in Figure 1. The search also identified systematic reviews evaluating other neuromodulation techniques, including non-invasive modalities. However, these reviews did not meet the eligibility criteria for the present umbrella review and were therefore excluded, as documented in Supplementary Table S3. Consequently, implanted ONS was the only neuromodulation modality represented in the final evidence synthesis.

**Figure 1:**
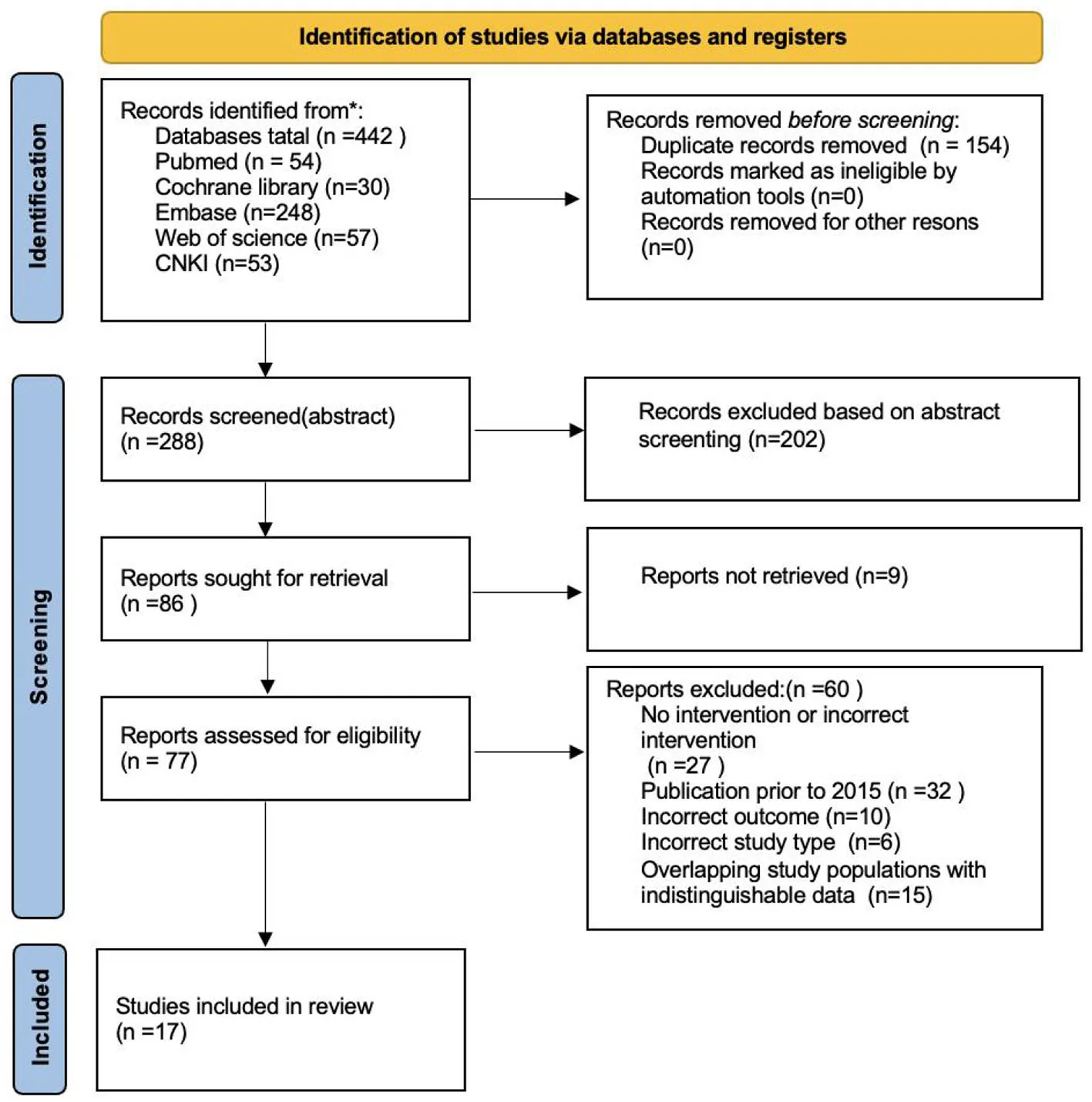
PRISMA flowchart.

### 3.2 Grading of evidence results (AMSTAR 2)

AMSTAR 2 assessment showed that 2 (26, 39) reviews (11.8%) were of high methodological quality, 5 (29, 30, 35, 37, 42) (29.4%) were of moderate quality, 7 (27, 28, 32, 34, 36, 38, 40) (41.2%) were of low quality, and 3 (31, 33, 41) (17.6%) were of critically low quality. The most common limitations were failure to provide a list of excluded studies with reasons, inadequate reporting of funding sources for primary studies, and insufficient assessment of small-study effects (Figure 2). Additional details are provided in Supplementary Table S4.

**Figure 2:**
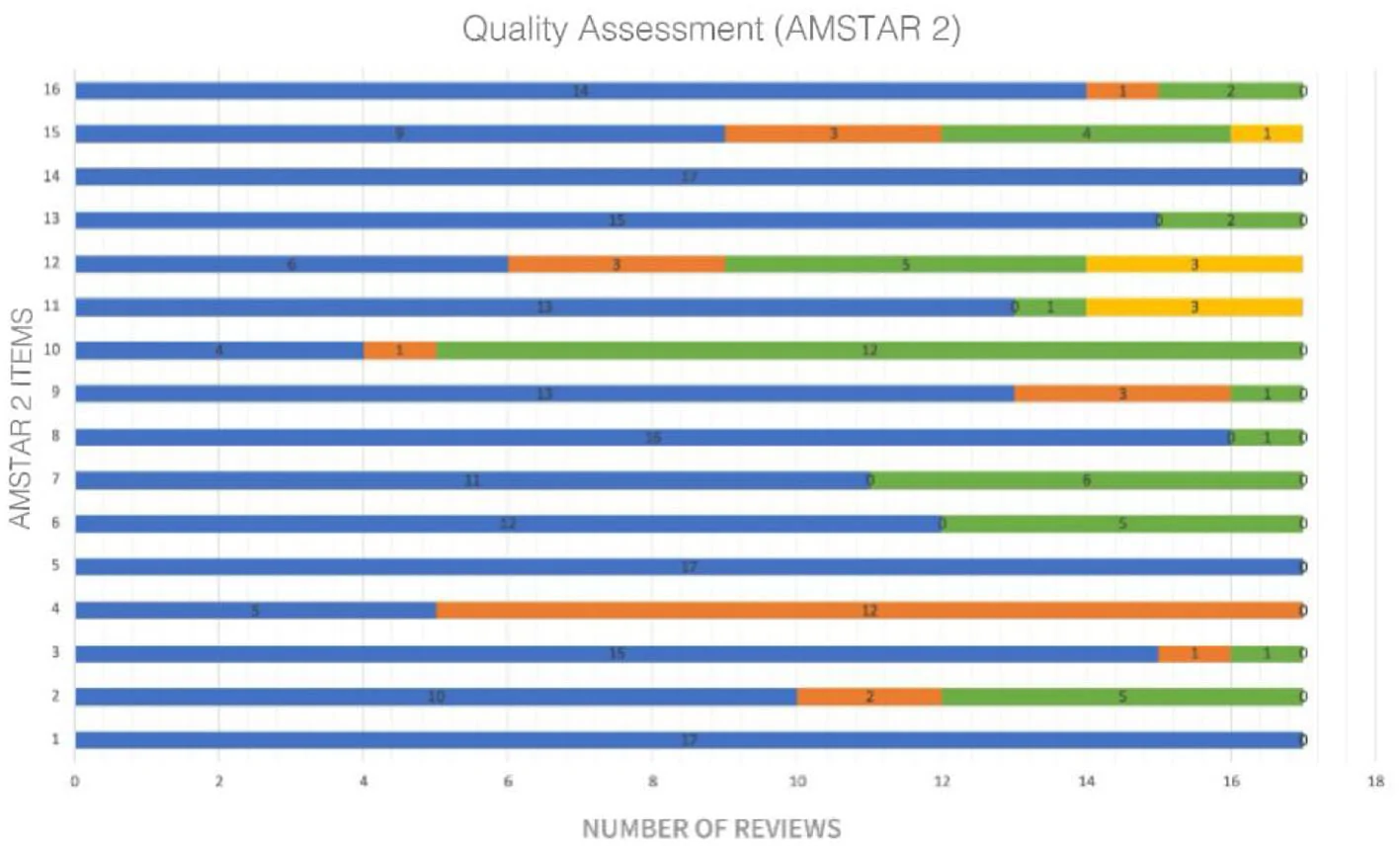
Methodological quality of included systematic reviews assessed using AMSTAR 2.

### 3.3 Grading of evidence results (GRADE)

GRADE assessment indicated that the certainty of evidence for most outcomes was low or very low. Downgrading was mainly driven by risk of bias, small sample sizes, imprecision, substantial heterogeneity, indirectness in populations or comparators, and insufficient assessment of publication bias. Details are provided in Supplementary Table S5.

### 3.4 Intervention-specific evidence synthesis

#### 3.4.1 OnabotulinumtoxinA

##### 3.4.1.1 Headache day

Two reviews reported standardized effects of onabotulinumtoxinA on headache day at different follow-up intervals. At 24 weeks, Barad et al. (26) reported a small reduction in headache day compared with sham (Hedges' g = −0.28; 95% CI, −0.41 to −0.15). Chen et al. (32) reported a larger standardized effect at 12 weeks (SMD = −0.58; 95% CI, −0.86 to −0.29). These review-level estimates were not re-pooled because they represented different follow-up intervals and overlap in the underlying PREEMPT trial populations could not be excluded. Although both estimates favored onabotulinumtoxinA, their standardized form prevents direct translation into the absolute number of headache day avoided.

Shaterian et al. (31) additionally summarized studies reporting reductions in headache day after onabotulinumtoxinA treatment. Several reports were related to the PREEMPT program and may have represented overlapping participant populations; these findings were therefore treated as contextual rather than independent quantitative evidence.

##### 3.4.1.2 Headache severity

Shaterian et al. (31) summarized several studies reporting reductions in headache severity after onabotulinumtoxinA treatment, including studies in adults with CM and pediatric populations. However, differences in patient populations, pain scales, dosing regimens, and injection protocols precluded estimation of a common effect. The available evidence therefore suggested possible improvement in headache severity but did not permit a reliable estimate of the magnitude or precision of the effect.

##### 3.4.1.3 HIT-6

Two reviews reported standardized effects on HIT-6 at different follow-up intervals. At 24 weeks, Barad (26) reported an improvement in HIT-6 with onabotulinumtoxinA compared with sham (Hedges' *g* = −0.36; 95% CI, −0.47 to −0.25). At 12 weeks, Chen et al. (32) reported an SMD of −0.44 (95% CI, −1.35 to 0.48); although the point estimate favored onabotulinumtoxinA, the confidence interval included the null value. The estimates were not re-pooled because the follow-up intervals differed and overlap in the underlying PREEMPT trial populations could not be excluded. Because both effects were expressed in standardized units, they cannot be directly compared with the ≥6-point within-person HIT-6 responder threshold, and the proportion of patients achieving a clinically meaningful HIT-6 response cannot be determined from these review-level estimates.

Shaterian et al. (31) additionally summarized PREEMPT-related reports showing reductions in HIT-6 and improvements in headache-related functioning. Because these publications may have included participants already represented in the quantitative evidence, they were treated as contextual rather than independent evidence.

##### 3.4.1.4 TRAEs

Two reviews provided comparative estimates of TRAEs for onabotulinumtoxinA vs. sham controls. Corasaniti et al. (29) reported a higher risk of TRAEs with onabotulinumtoxinA than with sham (RR = 2.29; 95% CI, 1.98 to 2.66), whereas Chen et al. (32) found no clear difference compared with placebo (RR = 1.03; 95% CI, 0.72 to 1.49). The two review-level estimates were not re-pooled because follow-up periods, TRAEs definitions, and underlying trial sets differed, and overlap in the included primary trials could not be excluded.

Shaterian et al. (31) described onabotulinumtoxinA as generally well tolerated but did not provide an independent pooled safety estimate. Reported events included neck pain, upper respiratory tract infection, migraine, and nasopharyngitis, whereas another small pediatric series reported no serious adverse events. Because event definitions, populations, comparators, and reporting periods varied across reports, these findings were summarized descriptively rather than combined into an overall safety estimate.

#### 3.4.2 Contextual evidence on adjunctive CGRP-targeted pharmacotherapy

One review (34) evaluated the addition of CGRP-targeted monoclonal antibody therapy to ongoing onabotulinumtoxinA treatment. This evidence was retained solely as clinical context because CGRP-targeted monoclonal antibodies are pharmacological preventive treatments rather than procedural interventions. The source review reported improvements from baseline in headache days, migraine-related disability, headache impact, and responder outcomes. However, all contributing cohorts were uncontrolled observational studies, and the estimates were affected by heterogeneity and incomplete outcome-level reporting. These findings are therefore preliminary and do not establish superiority over either component monotherapy. Detailed outcome-specific results are provided in Supplementary Text S1 and Supplementary Figure S1.

#### 3.4.3 Greater occipital nerve block

##### 3.4.3.1 Headache severity

At 4 weeks, GONB was associated with a reduction in headache severity compared with sham injection (MD = −1.33; 95% CI, −2.10 to −0.56; two RCTs; *n* = 116; *I*^2^ = 0%; Figure 3A). Although no statistical heterogeneity was observed, the estimate was based on only two trials, and the source review rated the evidence as low certainty. The clinical importance of the observed change could not be determined from the review-level estimate alone because a directly applicable scale-specific minimal clinically important difference was not available.

**Figure 3:**
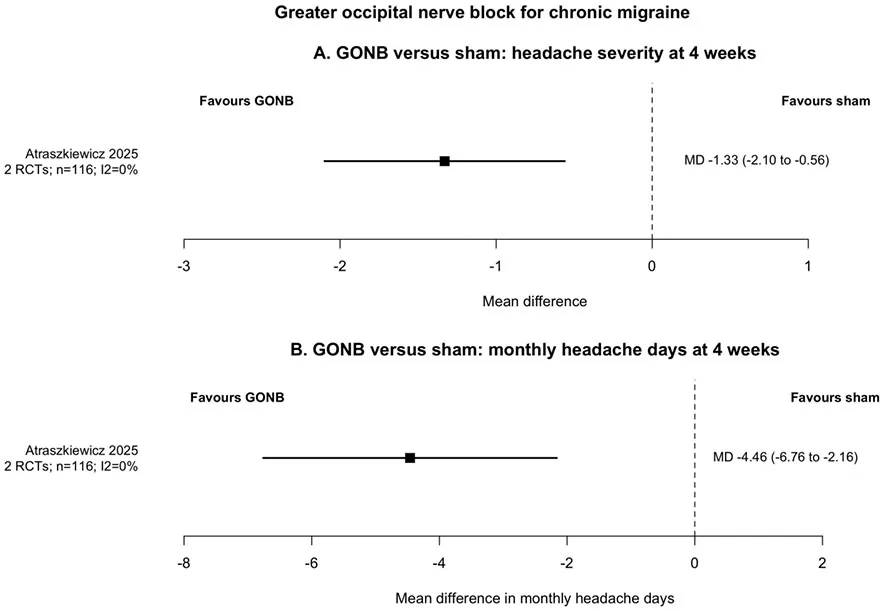
Intervention-specific evidence for greater occipital nerve block in CM. **(A)** Mean difference in headache severity at 4 weeks. **(B)** Mean difference in headache day at 4 weeks. Negative values favor GONB. Estimates were obtained from the clinically comparable randomized controlled trials synthesized by Atraszkiewicz (37).

The source review additionally reported variability across individual studies, with differences in treatment schedule, procedural technique, and follow-up period potentially contributing to inconsistent findings. These primary-study observations were considered contextual and were not treated as independent quantitative evidence.

##### 3.4.3.2 Headache day

Atraszkiewicz et al. (37) show that GONB was associated with approximately four fewer headache day than sham injection at 4 weeks (MD = −4.46 days; 95% CI, −6.76 to −2.16; two RCTs; *n* = 116; *I*^2^ = 0%; Figure 3B). This absolute between-group difference is clinically interpretable in headache day, although it is not directly equivalent to the proportion of individual patients achieving a ≥30% or ≥50% response. The source review rated the evidence as low certainty, and Chowdhury et al. (36) did not establish a consistent or sustained benefit across different injection schedules and follow-up intervals.

##### 3.4.3.3 Headache duration

Evidence for headache duration was inconsistent. Neither the open-label studies nor the placebo-controlled trials summarized in the source review demonstrated a consistent reduction in headache duration. Because reported improvements were primarily derived from uncontrolled or within-group comparisons, the available evidence did not establish a reliable treatment effect.

##### 3.4.3.4 MIDAS and HIT-6

Evidence for MIDAS and HIT-6 was derived mainly from uncontrolled observational studies, with some studies reporting improvements after repeated GONB. Because these findings lacked concurrent controls and were not quantitatively synthesized, they were considered exploratory and could not be reliably compared with established thresholds for clinically meaningful change.

##### 3.4.3.5 TRAEs

TRAEs were not quantitatively pooled. Reported events were predominantly mild and transient and included local injection-site pain, dizziness, nausea, vasovagal symptoms, and transient puncture-site discomfort. No serious adverse events were reported in the randomized trials. Because event definitions, denominators, injectates, and follow-up periods varied across studies, reliable pooled incidence estimates could not be derived. Corticosteroid-containing injections were additionally associated with potential local complications, including alopecia, hypopigmentation, and cutaneous atrophy.

#### 3.4.4 ONS

ONS was stratified according to study design, comparator, outcome, and effect measure. Controlled estimates comparing ONS with sham or subthreshold stimulation were reported separately from single-arm percentage changes from baseline. No additional second-order meta-analysis was conducted across reviews because the available estimates differed in comparator, analytical design, effect measure, and outcome definition. Review-level estimates from Barad et al. (26) were not displayed separately because the corresponding 12-week ONS outcomes were likely derived from the same underlying randomized trials and could therefore result in duplicate representation of the primary evidence.

##### 3.4.4.1 Headache day

Two studies (35) evaluated headache day at 12 weeks. Compared with sham or subthreshold stimulation, ONS was associated with a statistically significant reduction of approximately 3.06 headache day (MD = −3.06 days; 95% CI, −5.16 to −0.96; *P* = 0.004; *I*^2^ = 0%; Figure 4A). Although no statistical heterogeneity was observed, the estimate was based on only two studies and the source review rated the evidence as low certainty because of risk of bias and imprecision.

**Figure 4:**
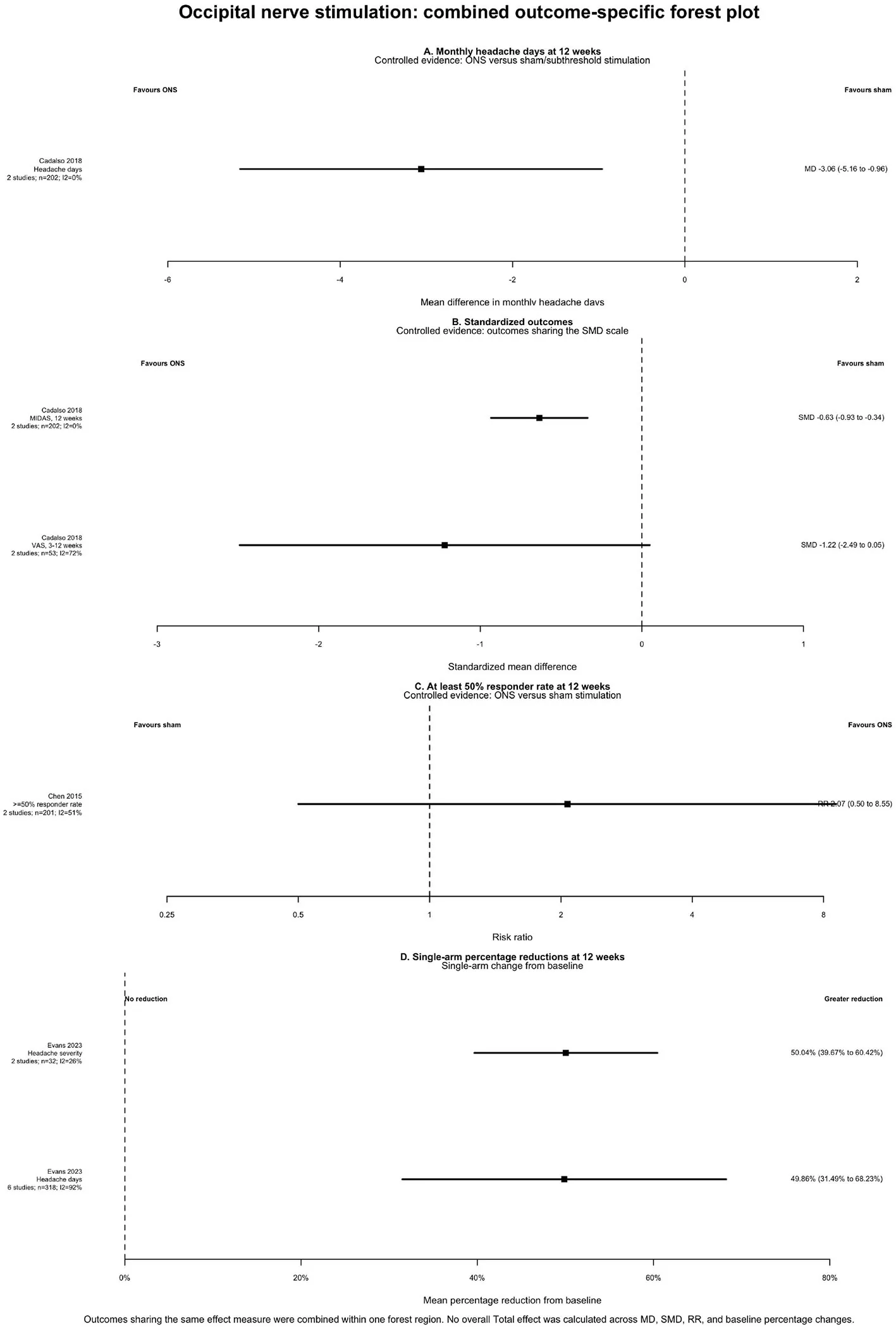
Intervention-specific evidence for occipital nerve stimulation. Controlled comparisons between ONS and sham or subthreshold stimulation are shown in panels **(A–D)**.

##### 3.4.4.2 MIDAS

Two studies (35) reported MIDAS at 12 weeks. ONS was associated with a statistically significant standardized improvement in migraine-related disability compared with sham stimulation (SMD = −0.63; 95% CI, −0.93 to −0.34; *P* < 0.001; *I*^2^ = 0%; Figure 4B). However, because the result was expressed as an SMD, it could not be translated into an absolute MIDAS-point reduction. It also could not be directly evaluated against baseline-dependent absolute or relative thresholds for clinically meaningful MIDAS improvement. The certainty of this evidence was low because of the small number of studies and risk of bias.

##### 3.4.4.3 Headache severity assessed using VAS

Two controlled studies (39) evaluated headache pain intensity over follow-up periods ranging from 3 to 12 weeks. ONS produced a numerically greater reduction in pain intensity than sham stimulation, but the difference did not reach statistical significance (SMD = −1.22; 95% CI, −2.49 to 0.05; *P* = 0.060; *I*^2^ = 72%; Figure 4C). The wide confidence interval and substantial heterogeneity indicate considerable uncertainty in both the presence and magnitude of the effect. Because the estimate was standardized, it could not be converted into an absolute VAS-point change or compared directly with a scale-specific minimal clinically important difference.

##### 3.4.4.4 At least 50% responder rate

Two studies reported (40) the proportion of patients achieving at least a 50% response at 12 weeks. ONS was not associated with a statistically significant increase in responder rate compared with sham stimulation (RR = 2.07; 95% CI, 0.50–8.55; *P* = 0.31; *I*^2^ = 51%; Figure 4D). The wide confidence interval included both no effect and a potentially large benefit, indicating substantial imprecision. In addition, responder definitions differed across the underlying trials, further limiting the clinical comparability of the pooled estimate. Therefore, the controlled evidence did not establish that ONS increased the likelihood of achieving a clinically meaningful individual response.

##### 3.4.4.5 Headache severity from baseline

In the single-arm analysis, two studies reported headache-severity outcomes at approximately 12 weeks. ONS was associated with a mean 50.04% reduction in headache severity from baseline (95% CI, 39.67%−60.42%; *I*^2^ = 26%). This estimate represents the pooled average percentage change from baseline rather than the proportion of patients achieving a 50% reduction. Because no concurrent control group was available, the improvement cannot be attributed exclusively to ONS and may also reflect regression to the mean, placebo effects, concomitant treatment, or natural variation in migraine severity.

##### 3.4.4.6 Headache day from baseline

Six studies evaluated the percentage change in headache day at approximately 12 weeks. ONS was associated with a pooled mean reduction of 49.86% from baseline (95% CI, 31.49%−68.23%; *P* < 0.00001; *I*^2^ = 92%). Given this high heterogeneity and the uncontrolled nature of the contributing studies, the pooled estimate was considered exploratory and should not be interpreted as a precise estimate of treatment effect.

Overall, controlled evidence suggested that ONS may reduce headache day and migraine-related disability in patients with refractory CM. In contrast, controlled effects on headache severity and the ≥50% responder rate remained statistically uncertain. Single-arm analyses also demonstrated substantial reductions from baseline in headache severity and headache day, but these findings lacked concurrent comparators and, for headache day, were characterized by considerable heterogeneity. No overall ONS effect was calculated because MDs, SMDs, RRs, and percentage changes represent different estimands and cannot be combined meaningfully. These findings should therefore be interpreted cautiously and primarily in the context of carefully selected patients with refractory CM.

#### 3.4.5 Acupuncture

##### 3.4.5.1 Effective rate

One review (27) evaluated a study-defined “effective rate” for acupuncture in patients with CM. Across 18 studies involving 1,412 participants, acupuncture was associated with higher odds of achieving the study-defined response than the control intervention (OR = 3.82; 95% CI, 2.88–5.06; *I*^2^ = 31%; Figure 5A). Statistical heterogeneity was low to moderate. However, the term “effective rate” was not based on a uniform internationally accepted CM outcome and may have incorporated different response definitions across the primary studies. In addition, the comparator and follow-up window were not clearly reported in the extracted review-level data. This result should therefore be interpreted as evidence of a favorable study-defined response rather than as a standardized ≥50% migraine responder outcome.

**Figure 5:**
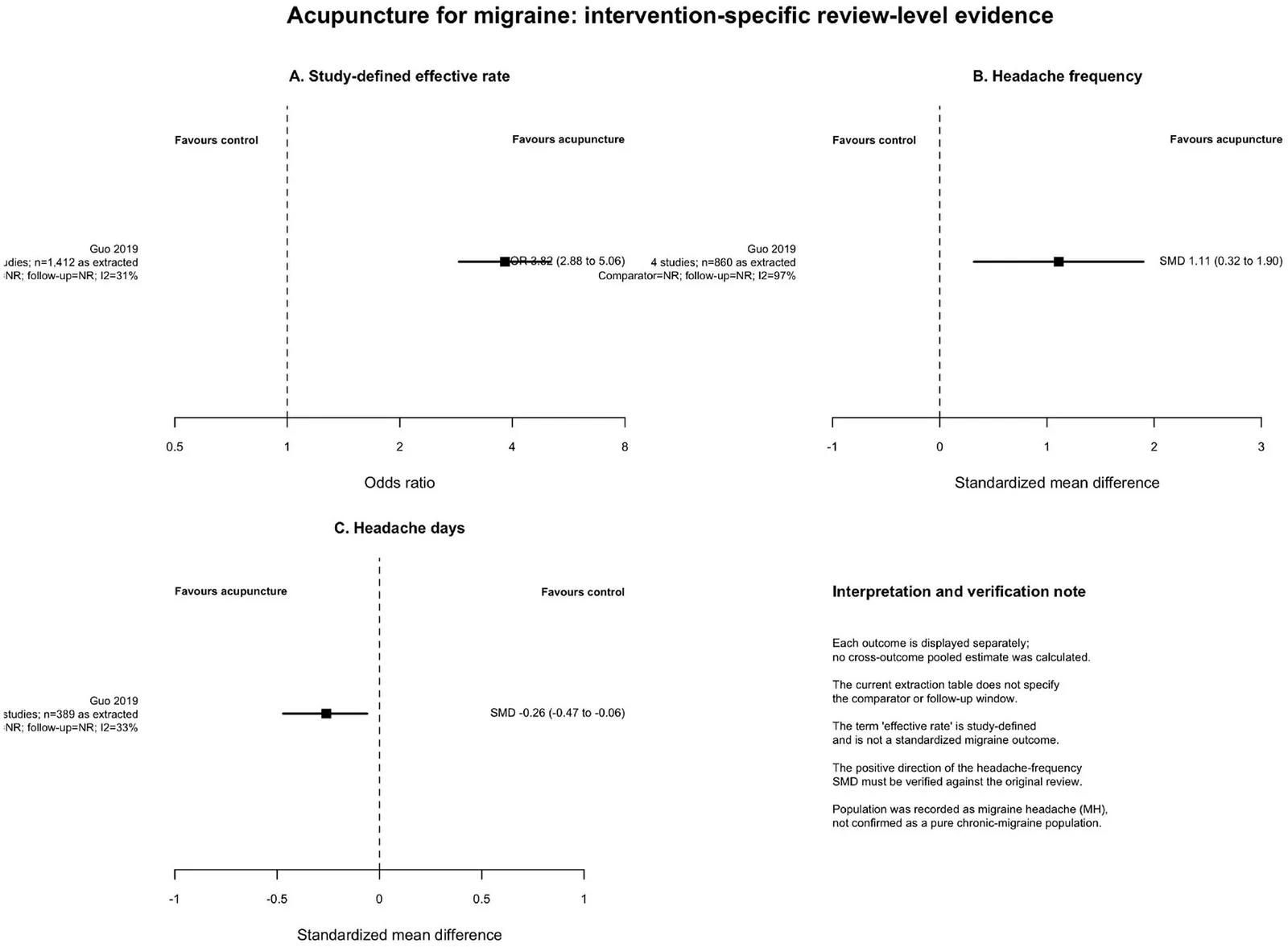
Intervention-specific review-level evidence for acupuncture in migraine. **(A)** Study-defined effective rate. **(B)** Headache frequency. **(C)** Headache day. Each outcome was displayed separately because the outcomes and effect measures were not clinically or statistically interchangeable.

##### 3.4.5.2 Headache frequency

Four studies involving 860 participants contributed to the analysis of headache frequency. The source review (27) reported an effect estimate in the direction coded as favoring acupuncture (SMD = 1.11; 95% CI, 0.32–1.90; Figure 5B). However, heterogeneity was considerable (*I*^2^ = 97%), indicating marked inconsistency in the magnitude of the effect across studies. Therefore, this pooled estimate was considered exploratory and should not be interpreted as a reliable estimate of the magnitude of benefit. The finding was used primarily to indicate the possible direction of effect.

##### 3.4.5.3 Headache day

Two studies (27) including 389 participants evaluated headache day. Acupuncture was associated with a small standardized reduction in headache day compared with the control intervention (SMD = −0.26; 95% CI, −0.47 to −0.06; *I*^2^ = 33%; Figure 5C). Statistical heterogeneity was low to moderate, and the confidence interval excluded the null value. However, the clinical relevance of this effect is uncertain because the standardized effect estimate cannot be directly translated into an absolute reduction in headache day or compared with established responder thresholds, such as a ≥30% or ≥50% reduction from baseline. The finding was also based on only two studies, limiting the certainty and generalizability of the evidence.

Overall, the review-level evidence suggested possible benefits of acupuncture for CM related outcomes; however, the findings were limited by inconsistent outcome definitions, incomplete reporting of comparators and follow-up periods, and substantial heterogeneity for headache frequency. The results should therefore be interpreted cautiously and should not be generalized directly to all patients with CM.

#### 3.4.6 Nerve block

##### 3.4.6.1 Headache severity

Two studies (41) including 195 participants evaluated changes in headache severity at 12 weeks. Nerve block treatment was associated with a reduction in headache severity scores from baseline (MD = −1.07; 95% CI, −1.79 to −0.36; *P* = 0.003; *I*^2^ = 0%; Figure 6A). However, the clinical importance of this change is uncertain because the pain scale and its minimal clinically important difference were not clearly reported in the extracted review-level data. Moreover, as this was a single-arm change-from-baseline analysis without a concurrent control group, the observed improvement cannot be attributed exclusively to the intervention.

**Figure 6:**
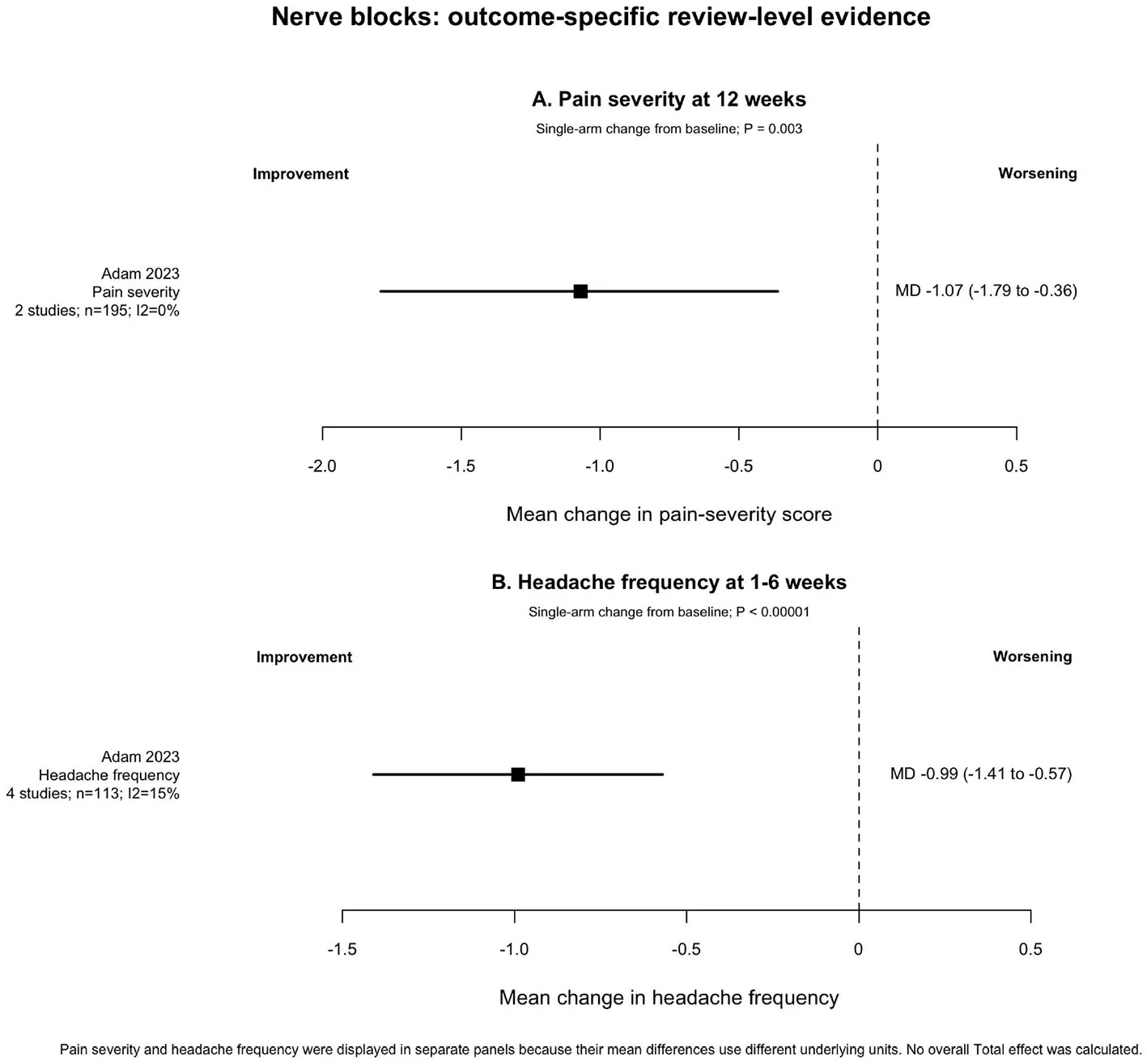
Outcome-specific review-level evidence for nerve block. **(A)** Mean change from baseline in headache severity at 12 weeks. **(B)** Mean change from baseline in headache frequency at 1–6 weeks. Negative values indicate improvement. Outcomes were displayed separately because their mean differences were measured using different underlying units.

##### 3.4.6.2 Headache frequency

Four studies (41) including 113 participants reported headache-frequency outcomes over follow-up periods ranging from 1 to 6 weeks. Nerve-block treatment was associated with a reduction in headache frequency from baseline (MD = −0.99; 95% CI, −1.41 to −0.57; *P* < 0.00001; *I*^2^ = 15%; Figure 6B). Although statistical heterogeneity was low, the pooled estimate combined measurements obtained at different short-term follow-up intervals, which may limit comparability across studies. The clinical relevance of the observed effect is also uncertain because the underlying frequency metric was not clearly defined in the extracted review-level data and may have represented headache attacks, headache day per week, or another measure. Accordingly, the estimate cannot be directly translated into a reduction in headache day or compared with established within-person responder thresholds, such as a ≥30% or ≥50% reduction from baseline.

##### 3.4.6.3 TRAEs

TRAEs reporting was inconsistent across the included trials (41), and no pooled safety estimate was available. Reported events included injection-site bleeding, local pain or tenderness, dizziness, neck and shoulder discomfort, transient sedation, rash, swelling, headache, vomiting, presyncope, and transient stinging at the puncture site. The source review described predominantly transient adverse events; however, event definitions and reporting denominators varied across studies, and no reliable pooled incidence estimate was available. Individual-study safety findings were therefore summarized descriptively and treated as contextual.

Overall, the review-level evidence suggested short-term improvements from baseline in both headache severity and headache frequency following nerve-block treatment. Nevertheless, the evidence was based on small numbers of studies and participants, lacked concurrent control groups, and incompletely reported the outcome scales and intervention protocols. These findings should therefore be interpreted as preliminary associations rather than evidence of superiority over sham procedures, usual care, or pharmacological treatment.

#### 3.4.7 Surgical interventions

Eighteen reports (42) derived from 17 distinct primary studies evaluated surgical interventions for CM, comprising 14 case series and four reports arising from three prospective randomized controlled trials. The procedures were clinically heterogeneous and included peripheral nerve decompression through myectomy or foraminotomy, nerve excision, arterial resection, and nasal surgery. In several studies, patients underwent individualized combinations of multiple procedures according to the presumed trigger sites. Because of incomplete baseline reporting, substantial intervention heterogeneity, and inconsistent outcome definitions, the findings were synthesized narratively, and no formal meta-analysis or additional umbrella-level pooled estimate was performed.

##### 3.4.7.1 Headache severity

According to the source review (42), migraine intensity was reported in 13 studies, most commonly using a 0–10 visual analog scale. Across the available studies, the descriptive mean migraine-intensity score decreased from 8.0 before surgery to 3.9 after surgery. Although all included studies reported some postoperative improvement, the magnitude of benefit varied considerably, and the descriptive pre–post averages were not adjusted for concurrent controls, regression to the mean, or differences in follow-up duration.

##### 3.4.7.2 Headache frequency

The source review (42) narratively summarized migraine-frequency outcomes from 11 studies. The descriptive average decreased from 15.6 migraine attacks or headache episodes per month before surgery to 5.1 per month after surgery. However, frequency definitions and assessment periods differed across studies, and the result was not derived from a comparative meta-analysis. Accordingly, the apparent reduction should be interpreted as a descriptive postoperative change rather than a causal between-group treatment effect.

##### 3.4.7.3 Headache duration

Eight studies (42) reported migraine duration. The descriptive mean duration decreased from 0.9 days before surgery to 0.4 days after surgery. Interpretation was limited by variation in the definition and recording of attack duration, incomplete reporting of dispersion measures, and differences in the surgical procedures and follow-up intervals.

##### 3.4.7.4 TREAs

TRAEs reporting was incomplete and inconsistent. Only 10 of the 18 included study reports provided safety information; seven reported variable postoperative complication rates, whereas three reported no adverse events (42). The descriptive overall TRAEs rate was 11.6%, with individual study estimates ranging from 0% to 38%. Reported complications included numbness or altered sensation, pruritus, alopecia, asymmetric brow elevation or ptosis, hematoma, paresthesia, nerve injury, neck stiffness or weakness, incisional cellulitis, hypertrophic scarring, and wound dehiscence. Because TRAEs definitions, reporting periods, surgical techniques, and denominators differed across studies, individual complications were not quantitatively pooled.

### 3.5 Primary-study overlap across included reviews

Across the 17 included reviews, the citation matrix contained 238 primary-study occurrences representing 202 unique primary studies, yielding an overall corrected covered area (CCA) of 1.11%, indicating slight overall overlap. However, overlap was not uniformly distributed across intervention categories and remained relevant within specific intervention–outcome domains, particularly among reviews containing the same pivotal studies. Because the overall CCA does not capture multiple publications, repeated follow-up assessments, or correlated outcomes derived from the same participant cohorts, overlapping reviews were not treated as independent confirmations of treatment effect or automatically re-pooled.

### 3.6 Intervention-specific evidence map

The intervention-specific evidence map integrated evidence volume, direction of benefit, AMSTAR 2, GRADE, and clinical position (Figure 7). OnabotulinumtoxinA had the most mature evidence base, supported by multiple systematic reviews and meta-analyses, a generally favorable efficacy signal, and an established role in CM prevention. GONB showed a short-term benefit signal for selected protocol-specific outcomes, but the certainty of evidence remained low.

**Figure 7:**
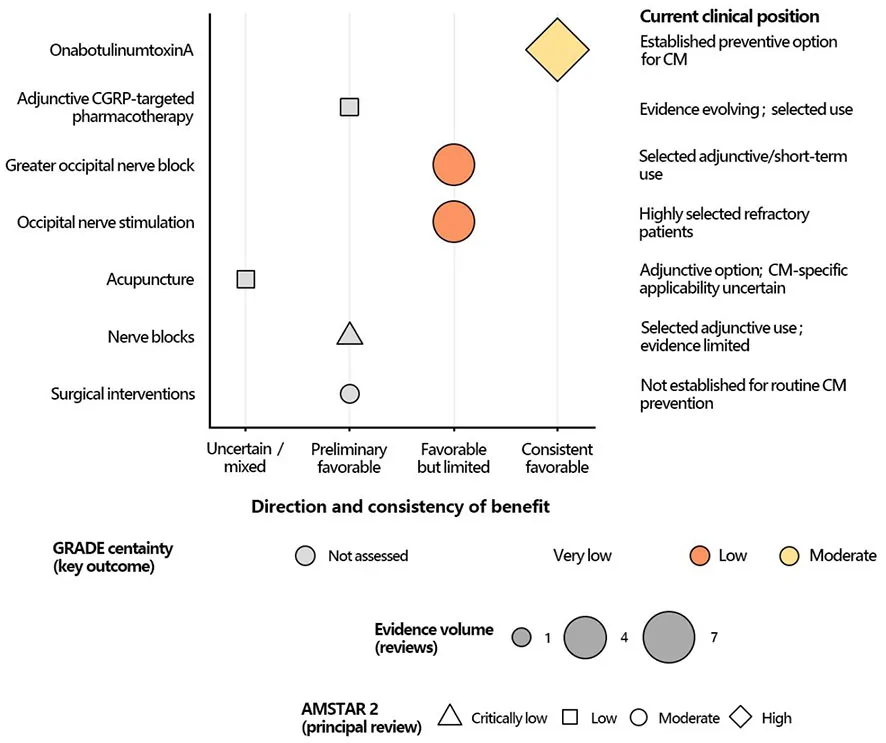
Intervention-specific evidence map for procedural interventions in chronic migraine. Evidence volume, direction of benefit, AMSTAR 2 methodological quality, GRADE certainty, and clinical context are summarized for each intervention category. Evidence concerning adjunctive CGRP-targeted monoclonal antibody therapy in patients receiving onabotulinumtoxinA is presented separately as contextual pharmacological evidence and is not classified as an independent procedural intervention.

Evidence for ONS, peripheral nerve blocks, acupuncture, and surgical interventions was more limited, heterogeneous, or based predominantly on observational or low-certainty evidence. Evidence concerning adjunctive CGRP-targeted monoclonal antibody therapy in patients receiving onabotulinumtoxinA was presented separately as contextual pharmacological evidence. AMSTAR 2 and GRADE were displayed separately because they assess different domains: methodological quality of the review and certainty of the underlying body of evidence, respectively. The evidence map was intended to summarize the maturity and limitations of the evidence rather than to provide a comparative ranking of treatment efficacy.

## 4 Discussion

### 4.1 Principal findings

This umbrella review shows that procedural interventions for CM should not be interpreted as a single therapeutic class. The included interventions differ substantially in mechanism, invasiveness, treatment setting, patient selection, safety profile, and maturity of evidence. OnabotulinumtoxinA had the most established evidence base and the clearest clinical role. GONB showed a protocol-specific short-term benefit signal, although certainty remained low and sustained benefit was not established. Evidence for ONS, acupuncture, other nerve blocks, and surgical interventions was more limited, heterogeneous, or restricted to selected populations. Separately, evidence concerning adjunctive CGRP-targeted monoclonal antibody therapy in patients receiving onabotulinumtoxinA was derived predominantly from uncontrolled observational cohorts and was therefore considered contextual and hypothesis-generating rather than evidence for an additional procedural intervention. Evidence volume did not necessarily correspond to evidence certainty. Some interventions were supported by multiple studies but remained dependent on uncontrolled designs, small samples, or heterogeneous protocols. Conversely, a methodologically rigorous systematic review could still yield low-certainty conclusions when the underlying primary studies were limited. This distinction supports separate consideration of AMSTAR 2, GRADE, direction of benefit, evidence volume, and clinical position rather than combining them into a single treatment ranking.

### 4.2 Interpretation of intervention-specific evidence

OnabotulinumtoxinA had the most mature evidence among the interventions examined. Its established role in CM prevention and the PREEMPT injection paradigm are supported by European Headache Federation guidance (43). Nevertheless, review-level estimates should be interpreted with attention to follow-up duration, outcome definition, and overlap among PREEMPT-related publications. Evidence supporting onabotulinumtoxinA should not be extrapolated to other procedural interventions with different mechanisms and evidentiary bases. GONB illustrates the importance of protocol-specific interpretation. Atraszkiewicz et al. (37) identified substantial methodological problems in previous syntheses, including pooling across non-equivalent injectates, schedules, laterality, co-interventions, outcomes, and follow-up intervals. In clinically comparable trials, weekly bilateral local-anesthetic GONB was associated with short-term reductions in headache severity and headache day, but the evidence was based on only two randomized trials and was rated as low certainty. GONB should therefore be considered a potentially useful short-term or adjunctive procedure rather than an established long-term preventive strategy. Adjunctive CGRP-targeted monoclonal antibody therapy has also been evaluated in patients already receiving onabotulinumtoxinA; however, the available evidence is derived from uncontrolled observational cohorts and is presented only as contextual pharmacological evidence rather than as evidence for an additional procedural intervention. These findings are therefore hypothesis-generating and do not establish superiority over either component alone. Controlled evidence for implanted ONS suggested possible improvements in headache day and migraine-related disability, whereas effects on headache severity and ≥50% responder outcomes remained uncertain. Single-arm studies reported larger improvements but were more susceptible to regression to the mean and other uncontrolled effects. The 2025 IHS guideline (7) addresses non-invasive neuromodulation and should not be interpreted as a direct recommendation for implanted ONS. Given its surgical and hardware-related risks, ONS remains most relevant to carefully selected patients with refractory CM.

### 4.3 Clinical positioning and patient selection

The clinical role of procedural therapies should be considered within the contemporary preventive-treatment landscape. CGRP-targeted therapies are now recognized as first-line preventive options, while onabotulinumtoxinA remains an established treatment for CM (44). OnabotulinumtoxinA has the clearest role in patients with established CM who require ongoing preventive treatment, particularly when headache burden remains substantial despite previous preventive management or when oral preventive therapies are poorly tolerated or unsuitable (45). Its standardized injection paradigm and comparatively mature evidence support repeated use when long-term prevention is needed (46). GONB may be considered when a short-term or adjunctive reduction in headache burden is required, although sustained benefit remains uncertain (47). As contextual pharmacological evidence, combined onabotulinumtoxinA and CGRP-targeted therapy has been evaluated in selected patients with an incomplete response to monotherapy; however, the available evidence remains predominantly observational and does not establish comparative superiority (48). Given its invasiveness and device-related risks, ONS should be reserved for highly refractory CM after established preventive options have failed or are unsuitable (49). Evidence for acupuncture, other nerve blocks, and surgery remains insufficient to define a routine role in CM.

### 4.4 Clinical relevance and benefit–risk considerations

Statistical significance should not be equated with clinical importance. Clinical interpretation should consider effect magnitude, responder outcomes, follow-up duration, baseline disease burden, and certainty of evidence rather than statistical estimates alone. Benefit–risk profiles also differ substantially across procedures. The degree and nature of invasiveness vary considerably, from repeated injections and percutaneous needling to surgically implanted stimulation devices and operative procedures. These differences have practical implications for patient selection, procedural burden, reversibility, recovery, and the management of treatment-related complications. OnabotulinumtoxinA has the most mature preventive evidence and a comparatively well-characterized safety profile, although repeated injections and treatment burden should be considered. GONB is less invasive and is generally associated with mild, transient adverse events; however, these advantages must be weighed against low-certainty evidence and uncertain durability of benefit. For ONS, potential benefit in refractory CM must be balanced against device- and surgery-related complications and the burden of implantation. Surgical interventions entail additional operative risks, while the certainty of benefit remains very low. Accordingly, procedural selection should be based on the overall balance of expected benefit, evidence certainty, safety, invasiveness, and durability rather than efficacy alone.

### 4.5 Economic and resource considerations

Economic and resource considerations are relevant to real-world procedural decision-making. However, health-economic outcomes were not prespecified or systematically extracted in this umbrella review and were therefore not formally synthesized. Available economic evidence remains limited and is highly context dependent, reflecting differences in healthcare systems, procedural and administration costs, treatment frequency, durability of benefit, and reimbursement structures. Accordingly, cost-effectiveness should not be inferred from clinical efficacy or per-procedure cost alone. Further comparative health-economic evaluations are needed to clarify the value of procedural interventions in routine CM care.

### 4.6 Methodological implications

Several methodological considerations emerged from this review. Clinical comparability should be assessed before statistical heterogeneity. A low *I*^2^ value does not establish that studies are clinically interchangeable, and a random-effects model cannot correct for incompatible treatment protocols, comparators, outcome definitions, or follow-up periods. Future syntheses should therefore prespecify intervention–outcome–time-point questions and avoid pooling studies solely because they share a broad procedural label.

Primary-study overlap also requires interpretation beyond a global CCA value. Although overall overlap was slight, duplication was concentrated within some intervention-specific evidence bases, and CCA does not fully capture multiple publications, repeated follow-up assessments, or correlated outcomes from the same cohort. Multiple reviews of the same pivotal trials should therefore not be treated as independent confirmation of efficacy.

Future studies should use clearly defined chronic-migraine populations, standardized procedural protocols, appropriate sham comparators, prespecified follow-up windows, and clinically interpretable outcomes. Consistent reporting of monthly headache or migraine days, responder rates, validated disability measures, adverse events, previous preventive failures, medication overuse, and comorbidities would improve both clinical applicability and future evidence synthesis.

### 4.7 Clinical heterogeneity

Clinical heterogeneity occurred both between different procedural interventions and within individual intervention categories. Even procedures sharing the same name were not necessarily delivered using equivalent protocols. For example, nerve-block studies differed in the choice of injectate, injection schedule, laterality, procedural technique, and use of concomitant treatments. Such variation may affect treatment effects, safety outcomes, and the comparability of findings across studies. Consequently, estimates derived from different protocols should not be assumed to represent a uniform treatment effect. Because only a small number of studies contributed to several intervention–outcome comparisons, formal subgroup or meta-regression analyses were generally not considered reliable. These potential sources of heterogeneity were therefore examined descriptively, and highly heterogeneous pooled estimates were interpreted cautiously or treated as exploratory.

## 5 Strengths and limitations

This review has several strengths. It used a preregistered umbrella-review framework, independent study selection, structured data extraction, AMSTAR 2 assessment of review methodology, GRADE assessment of evidence certainty, and citation-matrix analysis of primary-study overlap. The synthesis was organized by specific intervention rather than by a broad category of “invasive” or “procedural” treatment, and clinically dissimilar effect measures, comparators, treatment protocols, and follow-up periods were not combined solely to increase statistical power. The review also distinguished statistical significance from clinical relevance and integrated evidence volume, direction of benefit, methodological quality, certainty and clinical position in an intervention-specific evidence map.

Several limitations should be acknowledged. First, this umbrella review relied primarily on published review-level estimates and may therefore have inherited data-extraction errors, selective reporting, or analytical limitations from source reviews. Second, although overall primary-study overlap was slight, the CCA does not capture all dependencies arising from multiple publications, repeated follow-up assessments, or correlated outcomes from the same cohorts. Third, substantial clinical and methodological heterogeneity across reviews—including differences in populations, intervention protocols, comparators, outcomes, follow-up periods, and analytical methods—limited comparability and opportunities for valid secondary pooling. Fourth, several intervention–outcome questions were informed by few studies, small samples, observational designs, or highly selected populations, reducing precision and generalizability. Patient-level factors relevant to treatment selection were also inconsistently reported, limiting assessment of effect modification. In addition, standardized effects and mean changes were not always directly comparable with individual-level clinically meaningful thresholds. Finally, no head-to-head or network comparisons were performed; therefore, the evidence map reflects evidence maturity and clinical context rather than comparative treatment efficacy.

## 6 Conclusion

Procedural interventions for CM are clinically and methodologically distinct and should not be interpreted as a simple efficacy ranking. OnabotulinumtoxinA has the most established evidence and clinical role, whereas GONB may provide a short-term or adjunctive option with low-certainty evidence. ONS may be considered only in carefully selected patients with refractory CM, while evidence for other procedures remains limited or population-specific. Treatment selection should therefore consider evidence certainty, expected benefit, durability, safety, procedural burden, previous treatment response, and patient preference. Future studies should prioritize standardized protocols, clinically meaningful outcomes, long-term benefit–risk assessment, and health-economic evaluation.

## Data Availability

The original contributions presented in the study are included in the article/Supplementary material, further inquiries can be directed to the corresponding author.
