# Supplementary material for: Procedural interventions for chronic migraine: an umbrella review intervention-specific evidence map
Source: Front Neurol. 2026 Oct 12;17:1915292. doi: 10.3389/fneur.2026.1915292 (PMC13696246; doi:10.3389/fneur.2026.1915292)
Supplement: Supplementary file 1 [file Table_1.docx]

**Supplementary Table S1.** Search strategy

| **Database** | **Search term** | **Results** |
| --- | --- | --- |
| PubMed | ("Chronic Migraine"[MeSH Terms] OR "Chronic Migraine"[Title/Abstract] OR "Chronic Daily Headache"[Title/Abstract])AND("Nerve Block"[MeSH Terms] OR "Nerve Block"[Title/Abstract] OR "Botulinum Toxins, Type A"[MeSH Terms] OR "Botulinum Toxin A"[Title/Abstract] OR "Neurostimulation"[MeSH Terms] OR "Occipital Nerve Stimulation"[Title/Abstract] OR "Surgical Decompression"[MeSH Terms]OR "Surgical Decompression"[Title/Abstract] OR "Migraine Surgery"[Title/Abstract] OR "Radiofrequency Ablation"[MeSH Terms] OR "Radiofrequency Ablation"[Title/Abstract] OR "Acupuncture"[MeSH Terms] OR "Acupuncture"[Title/Abstract] OR "Electroacupuncture"[Title/Abstract] OR "Dry Needling"[Title/Abstract])AND(“Meta-Analysis” [pt] OR "Systematic Review"[pt] OR "Meta-Analysis"[Title/Abstract] OR "Systematic Review"[Title/Abstract]) | 54 |
| Web of Science | "Chronic Migraine" OR "Chronic Daily Headache" (Topic) and "Nerve Block" OR "Botulinum Toxins, Type A" OR "Botulinum Toxin A" OR "Neurostimulation" OR "Occipital Nerve Stimulation" OR "Surgical Decompression" OR "Migraine Surgery" OR "Radiofrequency Ablation" OR "Acupuncture" OR "Electroacupuncture" OR "Dry Needling" (Topic) and "Meta-Analysis" OR "Systematic Review" (Topic) | 57 |
| Embase | ('chronic migraine'/exp OR 'chronic migraine':ab,ti OR 'chronic daily headache':ab,ti)AND('nerve block'/exp OR 'nerve block':ab,ti OR 'botulinum toxin type a'/exp OR 'botulinum toxin a':ab,ti OR 'neurostimulation'/exp OR 'neurostimulation':ab,ti OR 'occipital nerve stimulation':ab,ti OR 'surgical decompression'/exp OR 'surgical decompression':ab,ti OR 'migraine surgery':ab,ti OR 'radiofrequency ablation'/exp OR 'radiofrequency ablation':ab,ti OR 'acupuncture'/exp OR 'acupuncture':ab,ti OR 'electroacupuncture'/exp OR 'electroacupuncture':ab,ti OR 'dry needling':ab,ti) AND (‘meta analysis'/exp OR 'meta analysis':ab,ti OR 'systematic review'/exp OR 'systematic review':ab,ti) | 248 |
| Cochrane Library | (MeSH descriptor: [Chronic Migraine] explode all trees OR (Chronic Migraine or Chronic Daily Headache):ti,ab,kw) AND (MeSH descriptor: [Nerve Block] explode all trees OR MeSH descriptor: [Botulinum Toxins, Type A] explode alli trees OR MeSH descriptor: [Neurostimulation] explode all trees OR MeSH descriptor: [Surgical Decompression] explode all trees OR MeSH descriptor: [Radiofrequency Ablation] explode all trees OR MeSH descriptor: [Acupuncture] explode all trees OR (Nerve Block or Botulinum Toxin A or Neurostimulation or Occipital Nerve Stimulation or Surgical Decompression or Migraine Surgery or Radiofrequency Ablation or Acupuncture or Electroacupuncture or Dry Needling):ti,ab,kw) AND (Meta-Analysis or Systematic Review):ti,ab,kw | 30 |
| CNKI | SU=('慢性偏头痛' + '慢性每日头痛') AND SU=('神经阻滞' + '肉毒毒素' + '肉毒毒素A' + '神经刺激' + '枕神经刺激' + '手术减压' + '偏头痛手术'+ '射频消融' + '针刺' + '针灸' + '电针' + '干针') AND SU=('系统综述' + '系统评价' + 'Meta分析' + '荟萃分析') | 53 |
| **TOTAL** |  | **442** |

**Supplementary Table S2.** Summary of associations of invasive therapy with CM.

| **Source** | **Procedural interventions** | **Comparator** | **Outcome** | **Follow-up（week）** | **Population** | **No. o s** | **Sample size** | **Metric** | **Random effect size (95% CI)** | ***P* value** | **I^2^, %** |
| --- | --- | --- | --- | --- | --- | --- | --- | --- | --- | --- | --- |
| Barad 2022 | OnabotulinumtoxinA | sham | Headache day | 24 | CM | 2 | 688/696 | SMD | −0.28（−0.41,−0.15） | <0.01 | NR |
| Barad 2022 | OnabotulinumtoxinA | sham | HIT-6 | 24 | CM | 2 | 688/697 | SMD | −0.36（−0.47, −0.25） | NR | NR |
| Barad 2022 | ONS | sham | Headache day | 12 | CM | 2 | 133/69 | SMD | −0.52（−0.81,−0.23） | <0.01 | NR |
| Barad 2022 | ONS | sham | MIDAS | 12 | CM | 2 | 133/69 | SMD | −0.58（−0.87,−0.28） | <0.01 | NR |
| Barad 2022 | GONB | sham | Headache day | 4 | CM | 2 | 55/36 | SMD | −0.87（−1.26,−0.48） | <0.01 | NR |
| Guo 2019 | Acupuncture | / | Effective rate | NR | MH | 18 | 1263/149 | OR | 3.82(2.88 to 5.06) | <0.00001 | 31 |
| Guo 2019 | Acupuncture | / | Headache frequency | NR | MH | 4 | 412/448 | SMD | 1.11(0.32 to 1.90) | 0.006 | 97 |
| Guo 2019 | Acupuncture | / | Headache day | NR | MH | 2 | 161/228 | SMD | -0.26(-0.47 to -0.06) | 0.01 | 33 |
| Lanteri-Mnet 2022 | OnabotulinumtoxinA | Baseline | Headache day | 24 | CM | 7 | 1750 | MD | -10.64 (-12.31 to -8.97) | <0.01 | NR |
| Lanteri-Mnet 2022 | OnabotulinumtoxinA | / | ≥50% reduction responder rate | 24 | CM | 7 | 1359 | Proportion | 46.57% (29.50% to 63.65%) | N/A | NR |
| Lanteri-Mnet 2022 | OnabotulinumtoxinA | / | MIDAS | 24 | CM | 4 | 425 | MD | 44.74 (28.50 to 60.99) | <0.00001 | 56 |
| Lanteri-Mnet 2022 | OnabotulinumtoxinA | Baseline | HIT-6 | 24 | CM | 1 | 211 | MD | -11.7 (-13.86 to -9.54) | <0.00001 | NR |
| Lanteri-Mnet 2022 | OnabotulinumtoxinA | Baseline | MSQ | 24 | CM | 1 | 972 | MD | 23.6 (21.56 to 25.64) | <0.00001 | NR |
| Corasaiti 2023 | OnabotulinumtoxinA | sham | TRAEs | Mixed, study-specific | CM | 7 | 3565 | RR | 1.31 (0.70 to 2.45) | 0.40 | 96 |
| Corasaiti 2023 | OnabotulinumtoxinA | sham | TRAEs | Mixed, approximately 12–56 | CM | 4 | 3169 | RR | 2.29 (1.98 to 2.66) | <0.00001 | 0 |
| Corasaiti 2023 | OnabotulinumtoxinA | medicine | TRAEs | Mixed, approximately 12–36 | CM | 3 | 396 | RR | 0.73 (0.23 to 2.34) | 0.59 | 96 |
| Lindsay 2024 | OnabotulinumtoxinA | Baseline | Headache Frequency | NR | CM | 5 | 170 | SMD | 0.97 (0.58 to 1.35) | <0.0001 | 66.09 |
| Lindsay 2024 | OnabotulinumtoxinA | Baseline | Headache Severity | NR | CM | 5 | 192 | SMD | 1.24 (0.55 to 1.94) | 0.0005 | 89.54 |
| Chen 2021 | OnabotulinumtoxinA | Placebo | Headache day | 12 | CM | 6 | 2809 | SMD | -0.58 (-0.86 to -0.29) | 0.80 | 83 |
| Chen 2021 | OnabotulinumtoxinA | Placebo | HIT-6 | 12 | CM | 4 | 1981 | SMD | -0.44 (-1.35 to 0.48) | 0.4 | 88.4 |
| Chen 2021 | OnabotulinumtoxinA | Placebo | MIDAS | 12 | CM | 3 | 1320 | SMD | -0.46 (-0.72 to -0.20) | 0.33 | 0 |
| Chen 2021 | OnabotulinumtoxinA | Placebo | TRAEs | 12 | CM | 5 | 2516 | RR | 1.03 (0.72 to 1.49) | 0.72 | 0 |
| Chen 2021 | OnabotulinumtoxinA | Placebo | Headache Frequency | NR | CM | 7 | 2912 | SMD | -0.43 (-0.70 to -0.17) | 0.001 | 0 |
| Liu 2020 | OnabotulinumtoxinA | Placebo | HIT-6 | NR | CM | 5 | 2526 | MD | -2.11 (-2.93 to -1.29) | <0.00001 | 4 |
| Liu 2020 | OnabotulinumtoxinA | Placebo | MIDAS | NR | CM | 5 | 2526 | MD | -2.11 (-2.93 to -1.29) | <0.00001 | 4 |
| Liu 2020 | OnabotulinumtoxinA | Placebo | TRAEs | NR | CM | 9 | 3419 | OR | 1.38 (1.20 to 1.58) | <0.00001 | 0 |
| Soltani 2026 | OnabotulinumtoxinA+ CGRP | Baseline | MIDAs | Mixed, approximately 4–36 | CM | 2 | 168 | MD | -47.38(-56.69 to -38.07) | <0.001 | NR |
| Soltani 2026 | OnabotulinumtoxinA+ CGRP | Baseline | HIT-6 | Mixed, approximately 4–36 | CM | 2 | 168 | MD | -8.21（-9.78 to -6.64） | <0.001 | NR |
| Soltani 2026 | OnabotulinumtoxinA+ CGRP | Baseline | ≥50% reduction responder rate | Mixed, approximately 4–36 | CM | 3 | NR | Proportion | 0.51 (0.44 to 0.58) | NR | NR |
| Soltani 2026 | OnabotulinumtoxinA+ CGRP | Baseline | ≥75% reduction responder rate | Mixed, approximately 4–36 | CM | 2 | NR | Proportion | 0.19 (0.14 to 0.26) | NR | NR |
| Soltani 2026 | OnabotulinumtoxinA+ CGRP | Baseline | Headache day | Mixed, approximately 4–36 | CM | 3 | NR | Proportion | -7.92 (-10.19 to -5.66) | NR | 75.8 |
| Cadalso 2018 | ONS | sham | VAS | Mixed, approximately 3–12 | CM | 2 | 53 | SMD | -1.220 (-2.489 to 0.049) | 0.060 | 72 |
| Cadalso 2018 | ONS | sham | MIDAs | 12 | CM | 2 | 202 | SMD | -0.634 (-0.933 to -0.335) | <0.001 | 0 |
| Cadalso 2018 | ONS | sham | Headache day | 12 | CM | 2 | 202 | MD | -3.061 (-5.162 to -0.961) | 0.004 | 0 |
| Atraszkiewcz 2025 | GONB | sham | Headache Severity | 4 | CM | 2 | 116 | MD | −1.33（−2.10 to −0.56） | <0.001 | 0 |
| Atraszkiewcz 2025 | GONB | sham | Headache day | 4 | CM | 2 | 116 | MD | −4.46 (−6.76 to −2.16） | <0.001 | 0 |
| Sabet 2025 | GONB | sham | Headache Severity | 4 | CM | 1 | 31 | MD | -3.57 (-3.95 to -3.19) | <0.00001 | 0 |
| Sabet 2025 | GONB | sham | Headache day | 4 | CM | 1 | 31 | MD | -12.12 (-13.95 to -10.29) | <0.00001 | 99 |
| Sabet 2025 | GONB | sham | headache duration | 4 | CM | 1 | 31 | MD | -11.51 (-14.61 to -8.41) | <0.00001 | 97 |
| Chen 2015 | ONS | sham | ≥50% reduction responder rate | 12 | CM | 2 | 201 | RR | 2.07 (0.50 to 8.55) | 0.31 | 51 |
| Evans 2023 | ONS | Baseline | Headache Severity | 12 | CM | 2 | 32 | Proportion | -0.5004 (-0.6042 to -0.3967) | NR | 26 |
| Evans 2023 | ONS | Baseline | Headache day | 12 | MD | 6 | 318 | Proportion | -0.4986 (-0.6823 to -0.3149) | <0.00001 | 92 |
| Adam 2023 | Nerve blocks | Baseline | Pain severity | 12 | MD | 2 | 195 | MD | -1.07(-1.79 to -0.36) | 0.003 | 0 |
| Adam 2023 | Nerve blocks | Baseline | Headache Frequency | 1-6 | MD | 4 | 113 | MD | -0.99(-1.41 to -0.57) | <0.00001 | 15 |

CM, Chronic Migraine; SMD, Standardized Mean Difference; MD, Mean Difference; OR, Odds Ratio; RR, Relative Risk; CI, Confidence Interval; ONS, occipital nerve stimulation; VAS, Headache intensity reduction; GONB, Greater Occipital Nerve Block; MIDAs, Migraine Disability Assessment Scale; HIT-6, Headache Impact Test-6; MSQ, Migraine-Specific Quality of Life Questionnaire;

**Supplementary Table S3.** Excluded studies with reasons from the search for meta-analyses of randomized controlled trials

| **Reasons for exclusion** | **References** |
| --- | --- |
| No intervention or incorrect intervention  (n=27) | 1. Clark O, Mahjoub A, Osman N, Surmava AM, Jan S, Lagman-Bartolome AM. Non-invasive neuromodulation in the acute treatment of migraine: a systematic review and meta-analysis of randomized controlled trials. Neurol Sci. 2022;43(1):153-165. doi:10.1007/s10072-021-05664-7 2. Chen YL, Chen Q, Li LW, Hua C, Zhang XY, Zheng H. Non-invasive brain stimulation treatments for migraine prophylaxis: a network meta-analysis of randomized controlled trials. Acta Neurol Belg. 2023;123(4):1481-1493. doi:10.1007/s13760-023-02277-z 3. Feng Y, Zhang B, Zhang J, Yin Y. Effects of Non-invasive Brain Stimulation on Headache Intensity and Frequency of Headache Attacks in Patients With Migraine: A Systematic Review and Meta-Analysis. Headache. 2019;59(9):1436-1447. doi:10.1111/head.13645 4. Huang YB, Yuan L, Xiao XY, Wang XY, Feng SJ, Zheng H. Effect of different non-pharmacologic placebo treatments on migraine prevention: a network meta-analysis of randomized controlled trials. Acta Neurol Belg. 2024;124(4):1125-1139. doi:10.1007/s13760-023-02460-2 5. Liu M, Yong Y, Zou H, Yang L, Qian J. The Short- and Long-Term Efficacy of Non-Invasive Brain Stimulation for Migraine: A Meta-Analysis. J Pain Res. 2025;18:4883-4900. Published 2025 Sep 19. doi:10.2147/JPR.S529622 6. Elwenspoek MM, Thom H, Sheppard AL, et al. Defining the optimum strategy for identifying adults and children with coeliac disease: systematic review and economic modelling. Health Technol Assess. 2022;26(44):1-310. doi:10.3310/ZUCE8371 7. Simmonds L, Lagrata S, Stubberud A, et al. An open-label observational study and meta-analysis of non-invasive vagus nerve stimulation in medically refractory chronic cluster headache. Front Neurol. 2023;14:1100426. Published 2023 Mar 30. doi:10.3389/fneur.2023.1100426 8. Moisset X, Pereira B, Ciampi de Andrade D, Fontaine D, Lantéri-Minet M, Mawet J. Neuromodulation techniques for acute and preventive migraine treatment: a systematic review and meta-analysis of randomized controlled trials. J Headache Pain. 2020;21(1):142. Published 2020 Dec 10. doi:10.1186/s10194-020-01204-4 9. Winter AC, Rice MS, Fortner RT, Eliassen AH, Kurth T, Tamimi RM. Migraine and breast cancer risk: a prospective cohort study and meta-analysis. J Natl Cancer Inst. 2014;107(1):381. Published 2014 Dec 12. doi:10.1093/jnci/dju381 10. Song D, Li P, Wang Y, Cao J. Noninvasive vagus nerve stimulation for migraine: a systematic review and meta-analysis of randomized controlled trials. Front Neurol. 2023;14:1190062. Published 2023 May 11. doi:10.3389/fneur.2023.1190062 11. de Coo IF, Marin JC, Silberstein SD, et al. Differential efficacy of non-invasive vagus nerve stimulation for the acute treatment of episodic and chronic cluster headache: A meta-analysis. Cephalalgia. 2019;39(8):967-977. doi:10.1177/0333102419856607 12. Pang Y, Cao T, Zhang Q, et al. Retinal microvasculature features in patients with migraine: a systematic review and meta-analysis. Front Neurol. 2023;14:1187559. Published 2023 Sep 15. doi:10.3389/fneur.2023.1187559 13. Kang J, Lee H, Yu S, et al. Effects and safety of transcranial direct current stimulation on multiple health outcomes: an umbrella review of randomized clinical trials. Mol Psychiatry. 2024;29(12):3789-3801. doi:10.1038/s41380-024-02624-3 14. Haghdoost F, Salam A, Seyed-Kolbadi FZ, Padala D, Delcourt C, Rodgers A. Transcranial Direct Current Stimulation in Episodic Migraine: A Systematic Review and Meta-Analysis of Randomized Controlled Trials. Med Sci (Basel). 2025;13(3):84. Published 2025 Jun 26. doi:10.3390/medsci13030084 15. Alnajjar AZ, Mustafa MMM, Abdelsalam OK, et al. Efficacy and safety of remote electrical neuromodulation in migraine: a comprehensive systematic review and meta-analysis. BMC Neurol. 2025;25(1):327. Published 2025 Aug 9. doi:10.1186/s12883-025-04291-5 16. Lan L, Zhang X, Li X, Rong X, Peng Y. The efficacy of transcranial magnetic stimulation on migraine: a meta-analysis of randomized controlled trails. J Headache Pain. 2017;18(1):86. Published 2017 Aug 22. doi:10.1186/s10194-017-0792 17. Schramm S, Börner C, Reichert M, et al. Perfusion imaging by arterial spin labeling in migraine: A literature review. J Cereb Blood Flow Metab. 2024;44(8):1253-1270. doi:10.1177/0271678X241237733 18. Tungushpayev M, Viderman D. Effects of Transcranial Direct Current Stimulation on pain and pain-related outcomes: an umbrella review. Korean J Pain. 2025;38(4):449-471. doi:10.3344/kjp.25019 19. Calabrò RS, Billeri L, Manuli A, Iacono A, Naro A. Applications of transcranial magnetic stimulation in migraine: evidence from a scoping review. J Integr Neurosci. 2022;21(4):110. doi:10.31083/j.jin2104110 20. Sabet H, Abbas A, Abouelmagd ME, et al. Ultrasound-guided greater occipital nerve block for chronic migraine: a systematic review and meta-analysis. Head Face Med. 2025;21(1):81. Published 2025 Nov 24. doi:10.1186/s13005-025-00554-1 21. Cheng YC, Zeng BY, Hung CM, et al. Effectiveness and acceptability of noninvasive brain and nerve stimulation techniques for migraine prophylaxis: a network meta-analysis of randomized controlled trials. J Headache Pain. 2022;23(1):28. Published 2022 Feb 20. doi:10.1186/s10194-022-01401-3 22. Ke D, Chen M, Dan X, et al. Effectiveness of neuromodulation techniques for migraine prophylaxis: a systematic review and network meta-analysis of randomized controlled trials. BMC Complement Med Ther. 2026;26(1):74. Published 2026 Jan 30. doi:10.1186/s12906-026-05270-0 23. Wang HZ, Wang WH, Shi HC, Yuan CH. Is there a reliable brain morphological signature for migraine?. J Headache Pain. 2020;21(1):89. Published 2020 Jul 11. doi:10.1186/s10194-020-01158-7 24. Tikka SK, Godi SM, Siddiqui MA, Garg S. Evidence from Indian studies on safety and efficacy of therapeutic transcranial magnetic stimulation across neuropsychiatric disorders- A systematic review and meta-analysis. Indian J Psychiatry. 2023;65(1):18-35. doi:10.4103/indianjpsychiatry.indianjpsychiatry_572_22 25. Pei JH, Ma T, Nan RL, et al. Mindfulness-Based Cognitive Therapy for Treating Chronic Pain A Systematic Review and Meta-analysis. Psychol Health Med. 2021;26(3):333-346. doi:10.1080/13548506.2020.1849746 26. Carrondo Cottin S, Gallani N, Cantin L, Prud'Homme M. Occipital nerve stimulation for non-migrainous chronic headaches: a systematic review protocol. Syst Rev. 2019;8(1):181. Published 2019 Jul 22. doi:10.1186/s13643-019-1101-x 27. Mojadidi MK, Winoker JS, Roberts SC, Msaouel P, Gevorgyan R, Zolty R. Two-dimensional echocardiography using second harmonic imaging for the diagnosis of intracardiac right-to-left shunt: a meta-analysis of prospective studies. Int J Cardiovasc Imaging. 2014;30(5):911-923. doi:10.1007/s10554-014-0426-8 |
| Publication prior to 2015 (n =32 ) | 1. Anttila V, Winsvold BS, Gormley P, et al. Genome-wide meta-analysis identifies new susceptibility loci for migraine. Nat Genet. 2013;45(8):912-917. doi:10.1038/ng.2676 2. Gibson LM, Whiteley W. The differential diagnosis of suspected stroke: a systematic review. J R Coll Physicians Edinb. 2013;43(2):114-118. doi:10.4997/JRCPE.2013.205 3. Xia W, Zhu M, Zhang Z, et al. Effect of Tianshu capsule in treatment of migraine: a meta-analysis of randomized control trials. J Tradit Chin Med. 2013;33(1):9-14. doi:10.1016/s0254-6272(13)60093-x 4. Huang Y, Cai X, Song X, et al. Steroids for preventing recurrence of acute severe migraine headaches: a meta-analysis. Eur J Neurol. 2013;20(8):1184-1190. doi:10.1111/ene.12155 5. Hu Y, Guan X, Fan L, Jin L. Triptans in prevention of menstrual migraine: a systematic review with meta-analysis. J Headache Pain. 2013;14(1):7. Published 2013 Jan 30. doi:10.1186/1129-2377-14-7 6. Yao G, Yu T, Han X, Mao X, Li B. Therapeutic effects and safety of olcegepant and telcagepant for migraine: A meta-analysis. Neural Regen Res. 2013;8(10):938-947. doi:10.3969/j.issn.1673-5374.2013.10.009 7. Harriott AM, Dueker N, Cheng YC, et al. Polymorphisms in migraine-associated gene, atp1a2, and ischemic stroke risk in a biracial population: the genetics of early onset stroke study. Springerplus. 2013;2(1):46. doi:10.1186/2193-1801-2-46 8. Sun H, Bastings E, Temeck J, et al. Migraine therapeutics in adolescents: a systematic analysis and historic perspectives of triptan trials in adolescents. JAMA Pediatr. 2013;167(3):243-249. doi:10.1001/jamapediatrics.2013.872 9. El-Chammas K, Keyes J, Thompson N, Vijayakumar J, Becher D, Jackson JL. Pharmacologic treatment of pediatric headaches: a meta-analysis. JAMA Pediatr. 2013;167(3):250-258. doi:10.1001/jamapediatrics.2013.508 10. Esserlind AL, Christensen AF, Le H, et al. Replication and meta-analysis of common variants identifies a genome-wide significant locus in migraine. Eur J Neurol. 2013;20(5):765-772. doi:10.1111/ene.12055 11. MacLean A, Sweeting H, Egan M, Der G, Adamson J, Hunt K. How robust is the evidence of an emerging or increasing female excess in physical morbidity between childhood and adolescence? Results of a systematic literature review and meta-analyses. Soc Sci Med. 2013;78:96-112. doi:10.1016/j.socscimed.2012.11.039 12. Brigo F, Storti M, Tezzon F, Manganotti P, Nardone R. Primary visual cortex excitability in migraine: a systematic review with meta-analysis. Neurol Sci. 2013;34(6):819-830. doi:10.1007/s10072-012-1274-8 13. Vos T, Flaxman AD, Naghavi M, et al. Years lived with disability (YLDs) for 1160 sequelae of 289 diseases and injuries 1990-2010: a systematic analysis for the Global Burden of Disease Study 2010. Lancet. 2012;380(9859):2163-2196. doi:10.1016/S0140-6736(12)61729-2 14. Balottin U, Fusar Poli P, Termine C, Molteni S, Galli F. Psychopathological symptoms in child and adolescent migraine and tension-type headache: a meta-analysis. Cephalalgia. 2013;33(2):112-122. doi:10.1177/0333102412468386 15. Davis D, Gregson J, Willeit P, Stephan B, Al-Shahi Salman R, Brayne C. Patent foramen ovale, ischemic stroke and migraine: systematic review and stratified meta-analysis of association studies. Neuroepidemiology. 2013;40(1):56-67. doi:10.1159/000341924 16. Miao J, Wang F, Fang Y. Association of 231G>A polymorphism of endothelin type A receptor gene with migraine: a meta-analysis. J Neurol Sci. 2012;323(1-2):232-235. doi:10.1016/j.jns.2012.09.027 17. Xun P, Qin B, Song Y, et al. Fish consumption and risk of stroke and its subtypes: accumulative evidence from a meta-analysis of prospective cohort studies. Eur J Clin Nutr. 2012;66(11):1199-1207. doi:10.1038/ejcn.2012.133 18. Pakpoor J, Handel AE, Giovannoni G, Dobson R, Ramagopalan SV. Meta-analysis of the relationship between multiple sclerosis and migraine [published correction appears in PLoS One. 2013;8(4). doi: 10.1371/annotation/274e5ef1-79aa-48f7-af62-0594d5c1354e]. PLoS One. 2012;7(9):e45295. doi:10.1371/journal.pone.0045295 19. Gu L, Yan Y, Long J, et al. The TNF-α-308G/A polymorphism is associated with migraine risk: A meta-analysis. Exp Ther Med. 2012;3(6):1082-1086. doi:10.3892/etm.2012.533 20. Guo Y, Han X, Yu T, Yao G. Meta-analysis of efficacy of topiramate in migraine prophylaxis. Neural Regen Res. 2012;7(23):1806-1811. doi:10.3969/j.issn.1673-5374.2012.23.007 21. Esserlind AL, Christensen AF, Le H, et al. Replication and meta-analysis of common variants identifies a genome-wide significant locus in migraine. Eur J Neurol. 2013;20(5):765-772. doi:10.1111/ene.12055 22. MacLean A, Sweeting H, Egan M, Der G, Adamson J, Hunt K. How robust is the evidence of an emerging or increasing female excess in physical morbidity between childhood and adolescence? Results of a systematic literature review and meta-analyses. Soc Sci Med. 2013;78:96-112. doi:10.1016/j.socscimed.2012.11.039 23. Brigo F, Storti M, Tezzon F, Manganotti P, Nardone R. Primary visual cortex excitability in migraine: a systematic review with meta-analysis. Neurol Sci. 2013;34(6):819-830. doi:10.1007/s10072-012-1274-8 24. Vos T, Flaxman AD, Naghavi M, et al. Years lived with disability (YLDs) for 1160 sequelae of 289 diseases and injuries 1990-2010: a systematic analysis for the Global Burden of Disease Study 2010. Lancet. 2012;380(9859):2163-2196. doi:10.1016/S0140-6736(12)61729-2 25. Balottin U, Fusar Poli P, Termine C, Molteni S, Galli F. Psychopathological symptoms in child and adolescent migraine and tension-type headache: a meta-analysis. Cephalalgia. 2013;33(2):112-122. doi:10.1177/0333102412468386 26. Davis D, Gregson J, Willeit P, Stephan B, Al-Shahi Salman R, Brayne C. Patent foramen ovale, ischemic stroke and migraine: systematic review and stratified meta-analysis of association studies. Neuroepidemiology. 2013;40(1):56-67. doi:10.1159/000341924 27. Miao J, Wang F, Fang Y. Association of 231G>A polymorphism of endothelin type A receptor gene with migraine: a meta-analysis. J Neurol Sci. 2012;323(1-2):232-235. doi:10.1016/j.jns.2012.09.027 28. Xun P, Qin B, Song Y, et al. Fish consumption and risk of stroke and its subtypes: accumulative evidence from a meta-analysis of prospective cohort studies. Eur J Clin Nutr. 2012;66(11):1199-1207. doi:10.1038/ejcn.2012.133 29. Pakpoor J, Handel AE, Giovannoni G, Dobson R, Ramagopalan SV. Meta-analysis of the relationship between multiple sclerosis and migraine [published correction appears in PLoS One. 2013;8(4). doi: 10.1371/annotation/274e5ef1-79aa-48f7-af62-0594d5c1354e]. PLoS One. 2012;7(9):e45295. doi:10.1371/journal.pone.0045295 30. Pinquart M, Shen Y. Depressive symptoms in children and adolescents with chronic physical illness: an updated meta-analysis. J Pediatr Psychol. 2011;36(4):375-384. doi:10.1093/jpepsy/jsq104 31. Gu L, Yan Y, Long J, et al. The TNF-α-308G/A polymorphism is associated with migraine risk: A meta-analysis. Exp Ther Med. 2012;3(6):1082-1086. doi:10.3892/etm.2012.533 32. Guo Y, Han X, Yu T, Yao G. Meta-analysis of efficacy of topiramate in migraine prophylaxis. Neural Regen Res. 2012;7(23):1806-1811. doi:10.3969/j.issn.1673-5374.2012.23.007 |
| Incorrect outcome (n=11) | 1. Schürks M, Rist PM, Zee RY, Chasman DI, Kurth T. Tumour necrosis factor gene polymorphisms and migraine: a systematic review and meta-analysis. Cephalalgia. 2011;31(13):1381-1404. doi:10.1177/0333102411419022 2. Schürks M, Rist PM, Zee RY, Chasman DI, Kurth T. Tumour necrosis factor gene polymorphisms and migraine: a systematic review and meta-analysis. Cephalalgia. 2011;31(13):1381-1404. doi:10.1177/0333102411419022 3. Pinquart M, Shen Y. Behavior problems in children and adolescents with chronic physical illness: a meta-analysis. J Pediatr Psychol. 2011;36(9):1003-1016. doi:10.1093/jpepsy/jsr042 4. Schürks M, Rist PM, Shapiro RE, Kurth T. Migraine and mortality: a systematic review and meta-analysis. Cephalalgia. 2011;31(12):1301-1314. doi:10.1177/0333102411415879 5. Cousins G, Hijazze S, Van de Laar FA, Fahey T. Diagnostic accuracy of the ID Migraine: a systematic review and meta-analysis. Headache. 2011;51(7):1140-1148. doi:10.1111/j.1526-4610.2011.01916.x 6. Rist PM, Diener HC, Kurth T, Schürks M. Migraine, migraine aura, and cervical artery dissection: a systematic review and meta-analysis. Cephalalgia. 2011;31(8):886-896. doi:10.1177/0333102411401634 7. Liu H, Liu M, Wang Y, et al. Association of 5-HTT gene polymorphisms with migraine: a systematic review and meta-analysis. J Neurol Sci. 2011;305(1-2):57-66. doi:10.1016/j.jns.2011.03.016 8. Pinquart M, Shen Y. Anxiety in children and adolescents with chronic physical illnesses: a meta-analysis. Acta Paediatr. 2011;100(8):1069-1076. doi:10.1111/j.1651-2227.2011.02223.x 9. Cole JW, Kittner SJ. Meta-analysis of results from case control and cohort studies finds that migraine is associated with approximately twice the risk of ischaemic stroke. Evid Based Med. 2010;15(6):193-194. doi:10.1136/ebm1146 10. Jackson JL, Shimeall W, Sessums L, et al. Tricyclic antidepressants and headaches: systematic review and meta-analysis. BMJ. 2010;341:c5222. Published 2010 Oct 20. doi:10.1136/bmj.c5222 11. Zaccara G, Perucca P, Loiacono G, Giovannelli F, Verrotti A. The adverse event profile of lacosamide: a systematic review and meta-analysis of randomized controlled trials. Epilepsia. 2013;54(1):66-74. doi:10.1111/j.1528-1167.2012.03589.x |
| Incorrect study type (n=6) | 1. Suthisisang CC, Poolsup N, Suksomboon N, Lertpipopmetha V, Tepwitukgid B. Meta-analysis of the efficacy and safety of naproxen sodium in the acute treatment of migraine. Headache. 2010;50(5):808-818. doi:10.1111/j.1526-4610.2010.01635.x 2. Schürks M, Rist PM, Kurth T. MTHFR 677C>T and ACE D/I polymorphisms in migraine: a systematic review and meta-analysis. Headache. 2010;50(4):588-599. doi:10.1111/j.1526-4610.2009.01570.x 3. Loke RWJ, Ortiz O, Gustin SM, et al. Convergent structural brain alterations in chronic pain: a multi-metric individual participant data meta-analysis. Brain Commun. 2026;8(3):fcag146. Published 2026 Apr 24. doi:10.1093/braincomms/fcag146 4. Khalili M, Haghdoost F, Liaghatdar A, et al. Effectiveness and Tolerability of Pharmacologic Prophylaxis for Chronic Migraine : A Systematic Review of Randomized Controlled Trials. Ann Intern Med. Published online May 5, 2026. doi:10.7326/ANNALS-25-02221 5. Lin X, Yu W, Lou J, Cao Z, Song W. The mechanism of different microglial receptors mediating central inflammation in chronic migraine: a meta-analysis. J Headache Pain. 2026;27(1):122. Published 2026 Apr 27. doi:10.1186/s10194-026-02372-5 6. Kumar Saxena A, Singh N, Chilkoti GT, Gulabani M, Kumar Malhotra R. Prevalence of chronic pain in the general population: A systematic review and meta-analysis. Indian J Med Res. 2026;163(3):355-370. doi:10.25259/IJMR_1935_2025 |
| Overlapping study populations with indistinguishable data (n=15) | 1. Wu J, Wang C, Meng F, Wang H. A systematic review and meta-analysis on the risk of migraine in patients with allergic rhinitis. Front Med (Lausanne). 2026;13:1766176. Published 2026 Apr 2. doi:10.3389/fmed.2026.1766176 2. Bruloy E, Sinna R, Grolleau JL, Bout-Roumazeilles A, Berard E, Chaput B. Botulinum Toxin versus Placebo: A Meta-Analysis of Prophylactic Treatment for Migraine. Plast Reconstr Surg. 2019 Jan;143(1):239-250. doi: 10.1097/PRS.0000000000005111. 3. Jackson JL, Kuriyama A, Hayashino Y. Botulinum toxin A for prophylactic treatment of migraine and tension headaches in adults: a meta-analysis. JAMA. 2012 Apr 25;307(16):1736-45. doi: 10.1001/jama.2012.505. 4. Atraszkiewicz D, Ünal E, Bassett P, Morell-Ducos F, Bahra A. Greater occipital nerve block for the treatment of migraine: An umbrella review, systematic review, and meta-analysis. Cephalalgia. 2025 Dec;45(12):3331024251398390. doi: 10.1177/03331024251398390. 5. Fornaro M, Di Lorenzo C, Steardo L Jr, et al. Prevalence and outcomes of co-occurring psychiatric and neurological disorders: An atlas based on umbrella review of 81 meta-analyses. Eur Neuropsychopharmacol. Published online March 26, 2026. doi:10.1016/j.euroneuro.2026.112820 6. Makita LM, Carolino GDGDS, Souza YG, et al. Anti-CGRP monoclonal antibodies for chronic migraine with medication-overuse headache: a conservative meta-analysis. Arq Neuropsiquiatr. 2026;84(3):1-9. doi:10.1055/s-0046-1817018 7. Sarvari Soltani A, Masoumi S, Soroush P, et al. Dual-mechanism preventive therapy in chronic migraine: an exploratory meta-analysis of onabotulinumtoxinA and anti-CGRP monoclonal antibody combination. Eur J Med Res. Published online February 20, 2026. doi:10.1186/s40001-026-04017-4 8. Cnockaert E, Meeus M, Cagnie B, et al. Human assumed central sensitization in interictal migraine: a systematic review and meta-analysis. Neurol Sci. 2026;47(3):250. Published 2026 Feb 11. doi:10.1007/s10072-026-08825-8 9. Carrondo Cottin S, Gallani N, Cantin L, Prud'Homme M. Occipital nerve stimulation for non-migrainous chronic headaches: a systematic review protocol. Syst Rev. 2019 Jul 22;8(1):181. doi: 10.1186/s13643-019-1101-x. 10. Velásquez-Rimachi V, Chachaima-Mar J, Cárdenas-Baltazar EC, Loayza-Vidalon A, Morán-Mariños C, Pacheco-Barrios K, Posso M, Alva-Díaz C. Greater occipital nerve block for chronic migraine patients: A meta-analysis. Acta Neurol Scand. 2022 Aug;146(2):101-114. doi: 10.1111/ane.13634. 11. Kollenburg L, Kurt E, Mulleners W, Abd-Elsayed A, Yazdi C, Schatman ME, Yong RJ, Cerda IH, Pappy A 2nd, Ashina S, Robinson CL, Dominguez M. Four Decades of Occipital Nerve Stimulation for Headache Disorders: A Systematic Review. Curr Pain Headache Rep. 2024 Oct;28(10):1015-1034. doi: 10.1007/s11916-024-01271-1. 12. Chamani G, Jasim H, Minston A, et al. Botulinum Toxin Type A for the Prevention of Migraines: An Umbrella Review of Systematic Reviews. Toxins (Basel). 2026;18(1):33. Published 2026 Jan 9. doi:10.3390/toxins18010033 13. Matharu MS, Silberstein S, Yuan H, et al. Migraine-related disability according to headache frequency subclassifications: A systematic review and meta-analysis. Cephalalgia. 2025;45(10):3331024251385965. doi:10.1177/03331024251385965 14. Khansa I, Janis JE. Discussion: Greater Occipital Nerve Block for the Treatment of Chronic Migraine Headaches: A Systematic Review and Meta-Analysis. Plast Reconstr Surg. 2019 Oct;144(4):953-955. doi: 10.1097/PRS.0000000000006060. 15. Shauly O, Gould DJ, Sahai-Srivastava S, Patel KM. Greater Occipital Nerve Block for the Treatment of Chronic Migraine Headaches: A Systematic Review and Meta-Analysis. Plast Reconstr Surg. 2019 Oct;144(4):943-952. doi: 10.1097/PRS.0000000000006059. |

**Supplementary Table S4.** AMSTAR2 results for individual studies included in the umbrella review

| **Study** | **1** | **2*** | **3** | **4*** | **5** | **6** | **7*** | **8** | **9*** | **10** | **11*** | **12** | **13*** | **14** | **15*** | **16** | **Overall confidence** |
| --- | --- | --- | --- | --- | --- | --- | --- | --- | --- | --- | --- | --- | --- | --- | --- | --- | --- |
| Barad 2022 | Y | Y | Y | PY | Y | Y | Y | Y | Y | PY | Y | Y | Y | Y | Y | Y | High |
| Guo 2019 | Y | PY | Y | PY | Y | Y | Y | Y | Y | N | N | Y | Y | Y | Y | Y | Low |
| Lanteri-Mnet 2022 | Y | N | Y | Y | Y | N | Y | Y | PY | Y | Y | PY | Y | Y | PY | Y | Low |
| Corasniti 2023 | Y | Y | Y | PY | Y | N | Y | Y | Y | N | Y | Y | Y | Y | Y | Y | Moderate |
| Lindsay 2024 | Y | Y | N | PY | Y | N | Y | Y | Y | N | Y | N | Y | Y | Y | Y | Moderate |
| Shaterian 2022 | Y | N | PY | PY | Y | Y | N | Y | N | N | N/A | N/A | N | Y | N/A | Y | Critically low |
| Chen 2021 | Y | Y | Y | Y | Y | Y | Y | Y | Y | N | Y | N | Y | Y | N | N | Low |
| Liu 2019 | Y | N | Y | PY | Y | Y | N | Y | Y | N | Y | N | Y | Y | Y | N | Critically low |
| Soltani 2026 | Y | Y | Y | PY | Y | N | N | Y | Y | N | Y | Y | Y | Y | Y | Y | Low |
| Robert 2018 | Y | PY | Y | PY | Y | Y | Y | N | Y | Y | Y | Y | Y | Y | Y | Y | Moderate |
| Chowdhury 2020 | Y | N | Y | PY | Y | N | Y | Y | Y | N | N/A | N/A | Y | Y | Y | Y | Low |
| Atraszkiewicz 2025 | Y | Y | Y | PY | Y | Y | Y | Y | Y | Y | Y | Y | Y | Y | N | Y | Moderate |
| Sabet 2025 | Y | N | Y | PY | Y | Y | N | Y | Y | N | Y | PY | Y | Y | N | Y | Low |
| Chen 2015 | Y | Y | Y | Y | Y | Y | Y | Y | Y | Y | Y | PY | Y | Y | PY | Y | High |
| Evans 2023 | Y | Y | Y | Y | Y | Y | N | Y | PY | N | Y | N | Y | Y | PY | PY | Low |
| Adam 2023 | Y | Y | Y | Y | Y | Y | N | Y | Y | N | Y | N | N | Y | N | Y | Critically low |
| Wormald 2019 | Y | Y | Y | PY | Y | Y | Y | Y | PY | N | N/A | N/A | Y | Y | Y | Y | Moderate |

Y, Yes; N, No; PY, Partially Yes; N/A, Not Applicable

**The definition of each item is as follows:**

1. Did the research questions and inclusion criteria for the review include the components of PICO?

2*. Did the report of the review contain an explicit statement that the review methods were established prior to the conduct of the review and did the report justify any significant deviations from the protocol?

3. Did the review authors explain their selection of the study designs for inclusion in the review?

4*. Did the review authors use a comprehensive literature search strategy?

5. Did the review authors perform study selection in duplicate?

6. Did the review authors perform data extraction in duplicate?

7*. Did the review authors provide a list of excluded studies and justify the exclusions?

8. Did the review authors describe the included studies in adequate detail?

9*. Did the review authors use a satisfactory technique for assessing the risk of bias (RoB) in individual studies that were included in the review?

10. Did the review authors report on the sources of funding for the studies included in the review?

11*. If meta-analysis was performed did the review authors use appropriate methods for statistical combination of results?

12. If meta-analysis was performed, did the review authors assess the potential impact of RoB in individual studies on the results of the meta-analysis or other evidence synthesis?

13*. Did the review authors account for RoB in individual studies when interpreting/ discussing the results of the review?

14. Did the review authors provide a satisfactory explanation for, and discussion of, any heterogeneity observed in the results of the review?

15*. If they performed quantitative synthesis did the review authors carry out an adequate investigation of publication bias small study bias and discuss its likely impact on the results of the review?

16. Did the review authors report any potential sources of conflict of interest, including any funding they received for conducting the review?

***Critical domain**

**The methodological quality evaluation criteria are as follows:**

**High**—No or one non-critical weakness: the systematic review provides an accurate and comprehensive summary of the results of the available studies that address the question of interest;

**Moderate**—More than one non-critical weakness: the systematic review has more than one weakness but no critical flaws. It may provide an accurate summary of the results of the available studies that were included in the review;

**Low**—One critical flaw with or without non-critical weaknesses: the review has a critical flaw and may not provide an accurate and comprehensive summary of the available studies that address the question of interest;

**Critically low**—More than one critical flaw with or without non-critical weaknesses: the review has more than one critical flaw and should not be relied on to provide an accurate and comprehensive summary of the available studies.

**Supplementary Table S5.** The results of GRADE assessment of the evidence certainty of Invasive therapy.

| **Study** | **Outcomes** | **Downgrade factors** | | | | | **Quality** |
| --- | --- | --- | --- | --- | --- | --- | --- |
|  |  | **Risk of bias** | **Imprecision** | **Inconsistency** | **Indirectness** | **Publication bias/P value** |  |

| Barad 2022 | OnabotulinumtoxinA: headache days | No serious | No serious | No serious | No serious | No | Moderate |
| --- | --- | --- | --- | --- | --- | --- | --- |
| Barad 2022 | OnabotulinumtoxinA: HIT-6 | No serious | No serious | No serious | No serious | No | Moderate |
| Barad 2022 | GONB: headache days | Serious a | Serious b | No serious | No serious | No | Very low |
| Barad 2022 | ONS: headache day | No serious | No serious | No serious | No serious | No | Moderate |
| Barad 2022 | ONS: MIDAS | No serious | No serious | No serious | No serious | No | Moderate |
| Guo 2019 | Acupuncture: Effective rate | Serious a | Serious b | Serious c | No serious | No | Low |
| Guo 2019 | Acupuncture: TRAEs | Serious a | No serious | No serious | Serious d | No | Moderate |
| Lanteri-Minet 2022 | OnabotulinumtoxinA: Headache day (at 24 weeks) | Serious a | No serious | Serious c | No serious | No | Very low |
| Lanteri-Minet 2022 | OnabotulinumtoxinA: ≥50% reduction in migraine days (at 24 weeks) | Serious a | Serious b | Serious c | No serious | No | Very low |
| Lanteri-Minet 2022 | OnabotulinumtoxinA: HIT-6 (at 24 weeks) | Serious a | Serious b | Not assessed | No serious | No | Very low |
| Lanteri-Minet 2022 | OnabotulinumtoxinA: MSQ (at 24 weeks) | Serious a | Serious b | Not assessed | No serious | No | Very low |
| Lanteri-Minet 2022 | OnabotulinumtoxinA: MIDAs (at 24 weeks) | Serious a | Serious b | Serious c | No serious | No | Very low |
| Corasaniti 2023 | OnabotulinumtoxinA: TRAEs | No serious | No serious | No serious | No serious | No | Moderate |
| Lindsay 2024 | OnabotulinumtoxinAZ: Headache frequency | Serious a | No serious | Serious c | No serious | No | Very low |
| Lindsay 2024 | OnabotulinumtoxinA: Headache severity | Serious a | Serious b | Serious c | No serious | No | Very low |
| Lindsay 2024 | OnabotulinumtoxinA: TRAEs | Serious a | Serious b | No serious | No serious | No | low |
| Shaterian 2022 | OnabotulinumtoxinA: Headache day | Serious a | Serious b | Serious c | Serious d | No | Low |
| Shaterian 2022 | OnabotulinumtoxinA:  Headache severity change | Serious a | Serious b | Serious c | Serious d | No | Low |
| Shaterian 2022 | OnabotulinumtoxinA:  HIT-6/MSQ | Serious a | Serious b | Serious c | Serious d | No | Low |
| Chen 2021 | OnabotulinumtoxinA: Headache day | No serious | Serious b | Moderate | Serious d | No | Moderate |
| Chen 2021 | OnabotulinumtoxinA: HIT-6/MSQ | No serious | Serious b | Moderate | Serious d | No | Moderate |
| Chen 2021 | OnabotulinumtoxinA: TRAEs | No serious | Serious b | No serious | Serious d | No | Moderate |
| Liu 2020 | OnabotulinumtoxinA: Headache frequency | No serious | No serious | No serious | No serious | No | High |
| Liu 2020 | OnabotulinumtoxinA: HIT-6 / MIDAS | No serious | Serious b | No serious | No serious | No | Moderate |
| Liu 2020 | OnabotulinumtoxinA: TRAEs | No serious | No serious | No serious | No serious | No | High |
| Soltani 2026 | OnabotulinumtoxinA: Headache day | Serious a | Serious b | Serious c | Serious d | No | Low |
| Soltani 2026 | OnabotulinumtoxinA: ≥50% responder rate | Serious a | Very Serious b | Moderate | Serious d | No | Low |
| Soltani 2026 | OnabotulinumtoxinA: ≥75% responder rate | Serious a | Very Serious b | Moderate | Serious d | No | Very Low |
| Soltani 2026 | OnabotulinumtoxinA: MIDAS/HIT-6 | Serious a | Very Serious b | Serious c | Serious d | No | Very Low |
| Cadalso 2018 | ONS: Responder rate | Serious a | Serious b | No serious | No serious | No | Low |
| Cadalso 2018 | ONS: Headache day | Serious a | Serious b | No serious | No serious | No | Low |
| Cadalso 2018 | ONS: VAS | Serious a | Serious b | Serious c | No serious | No | Very Low |
| Cadalso 2018 | ONS: MIDAs | Serious a | Serious b | No serious | No serious | No | Low |
| Chowdhury 2020 | GONB: Headache day | Serious a | Serious b | Serious c | Serious d | No | Low |
| Chowdhury 2020 | GONB: VAS | Serious a | Serious b | Serious c | Serious d | No | Low |
| Chowdhury 2020 | GONB: ≥50% responder rate | Serious a | Serious b | Serious c | Serious d | No | Low |
| Chowdhury 2020 | GONB: MIDAS / HIT-6 | Serious a | Serious b | Serious c | Serious d | No | Low |
| Atraszkiewicz 2025 | GONB: Headache severity | Serious a | No serious | No serious | Serious d | No | Low |
| Atraszkiewicz 2025 | GONB: Headache day | Serious a | No serious | No serious | Serious d | No | Low |
| Sabet 2025 | GONB: Headache severity | Serious a | Serious b | No serious | No serious | No | Low |
| Sabet 2025 | GONB: Headacheday | Serious a | Serious b | No serious | No serious | No | Low |
| Sabet 2025 | GONB: Headache duration | Serious a | Serious b | Serious c | No serious | Yea/P=0.89 | Very Low |
| Sabet 2025 | GONB: TRAEs | Serious a | Serious b | No serious | No serious | Not assessable | Low |
| Chen 2015 | ONS: Headache day | Serious a | Serious b | No serious | No serious | Yea/P=0.003 | Moderate |
| Chen 2015 | ONS: Responder rate | Serious a | Serious b | Moderate c | No serious | Yea/P=0.02 | Moderate |
| Chen 2015 | ONS: VAS | Serious a | Serious b | Serious c | No serious | No | Low |
| Chen 2015 | ONS: MIDAs | Serious a | Serious b | No serious | No serious | Yea/P=0.001 | Moderate |
| Evans 2023 | ONS: Headache severity | Serious a | Serious b | Serious c | No serious | No | Low |
| Evans 2023 | ONS: Headache frequency | Serious a | Serious b | Very Serious c | No serious | No | Very Low |
| Evans 2023 | ONS: TRAEs | Serious a | Serious b | Serious c | No serious | No | Very Low |
| Adam 2023 | Nerve blocks: Headache severity | Serious a | Serious b | No serious | Serious d | No | Low |
| Adam 2023 | Nerve blocks: Headache frequency | Serious a | Serious b | No serious | Serious d | No | Low |
| Adam 2023 | Nerve blocks: TRAEs | Serious a | Serious b | Serious c | Serious d | No | Very low |
| Wormald 2019 | Surgery: Headache severity | Serious a | Serious b | Serious c | Serious d | No | Very low |
| Wormald 2019 | Surgery: Headache frequency | Serious a | Serious b | Serious c | Serious d | No | Very low |
| Wormald 2019 | Surgery: Headache duration | Serious a | Serious b | Serious c | Serious d | No | Very low |
| Wormald 2019 | Surgery: TRAEs | Serious a | Serious b | Serious c | Serious d | No | Very low |

GRADE, Grading of Recommendations Assessment, Development and Evaluation; HIT-6, Headache Impact Test-6; MIDAS, Migraine Disability Assessment; MSQ , Migraine-Specific Quality of Life Questionnaire; TRAEs, Treatment-related adverse events; GONB, greater occipital nerve block; ONS, occipital nerve stimulation; CGRP, calcitonin gene-related peptide.

ᵃ Downgraded for risk of bias, because many included studies had unclear or inadequate reporting of random sequence generation, allocation concealment, blinding, adverse events, study limitations, and therapist qualifications.

^b^ Downgraded for imprecision, because the evidence was based on a small number of studies, or the confidence interval was wide and/or crossed the line of no effect.

^c^ Downgraded for inconsistency, because the overall meta-analysis showed moderate heterogeneity, with I² = 65%, and some outcomes were measured using inconsistent methods across studies.

^d^ Downgraded for indirectness, because carbamazepine is not a standard or commonly recommended treatment for migraine, making this comparator less directly relevant to routine clinical practice.

**Supplementary Text S1.** Detailed contextual evidence on adjunctive CGRP-targeted pharmacotherapy.

One review ^(34)^ evaluated the addition of CGRP-targeted monoclonal antibody therapy to ongoing onabotulinumtoxinA treatment and synthesized data from several observational cohorts. The estimates reported below were derived from the source meta-analysis and were not re-pooled at the umbrella-review level. All contributing cohorts were single-arm observational studies without a concurrent control group.

**Headache day**

The source review ^(34)^ pooled three observational cohorts reporting changes in monthly headache day. Combination therapy was associated with a mean reduction of 7.92 monthly headache day from baseline (MD = −7.92 days; 95% CI, −10.19 to −5.66; Supplementary Figure S1A). However, substantial statistical heterogeneity was observed (I² = 75.8%), indicating considerable variability in the magnitude of change across cohorts.. Because the estimate represented a single-arm change from baseline, it cannot be interpreted as evidence that combination therapy was superior to either onabotulinumtoxinA or CGRP-targeted monotherapy.

**MIDAS**

The source review ^(34)^ pooled data from two observational cohorts reporting changes in migraine-related disability using the MIDAS. The pooled mean MIDAS score decreased by 47.38 points from baseline following combination therapy (MD = −47.38; 95% CI, −56.69 to −38.07; Supplementary Figure S1B). Although the confidence interval did not cross zero, the clinical relevance of this change could not be formally assessed using baseline-dependent meaningful-change criteria because baseline MIDAS severity was not consistently reported. In particular, an absolute reduction threshold may be applicable to patients with lower baseline disability, whereas a relative reduction threshold is generally more appropriate for patients with higher baseline MIDAS scores. Therefore, the magnitude of the pooled mean change should not be interpreted as demonstrating that all patients achieved a clinically meaningful improvement.

**HIT-6**

The source review ^(34)^ pooled data from two observational cohorts reporting changes in HIT-6. Combination therapy was associated with a mean reduction of 8.21 points in HIT-6 score from baseline (MD = −8.21; 95% CI, −9.78 to −6.64; Supplementary Figure S1C). The magnitude of this pooled mean change was numerically greater than the commonly used 6-point within-person responder threshold for cohorts. CM. However, the pooled mean change and the within-person responder threshold represent different estimands and are not directly equivalent. Consequently, these data do not establish the proportion of individual patients who achieved a clinically meaningful HIT-6 response.

**At least 50% responder rate**

Three observational cohorts ^(34)^ contributed to the pooled ≥50% responder estimate reported in the source review. The pooled ≥50% responder proportion was 51% (95% CI, 44%–58%; Supplementary Figure S1D). This responder outcome is more directly clinically interpretable than a pooled mean change because it reflects the proportion of individual patients reaching a prespecified improvement threshold. Nevertheless, in the absence of a concurrent control group, the observed responder proportion cannot be attributed exclusively to the combination regimen and may also reflect continued effects of onabotulinumtoxinA, regression to the mean, natural variation in migraine frequency, concomitant treatments, or other time-related factors.

**At least 75% responder rate**

Two observational cohorts ^(34)^ contributed to the pooled ≥75% responder estimate reported in the source review. The pooled ≥75% responder proportion was 19% (95% CI, 14%–26%; Supplementary Figure S1E). This more stringent responder threshold indicates that approximately one in five patients experienced a marked reduction in headache frequency. However, the estimate was based on only two observational cohorts, and outcome-level sample sizes were not clearly reported. The precision and generalizability of this estimate therefore remain uncertain.

Overall, the findings suggest that combined onabotulinumtoxinA and CGRP-targeted monoclonal antibody therapy was associated with improvements from baseline in headache frequency, headache-related disability, and headache impact. However, all available estimates were derived from single-arm observational cohorts, follow-up durations varied across studies, and endpoint-level sample sizes were incompletely reported. The evidence should therefore be considered preliminary and hypothesis-generating and should not be interpreted as demonstrating superiority over either component monotherapy.

**Supplementary Figure S1. Contextual evidence on adjunctive CGRP-targeted monoclonal antibody therapy in patients receiving onabotulinumtoxinA.** (A) Change from baseline in monthly headache days. (B) Change from baseline in Migraine Disability Assessment (MIDAS) score. (C) Change from baseline in Headache Impact Test-6 (HIT-6) score. (D) Proportion of patients achieving at least a 50% reduction in headache frequency. (E) Proportion of patients achieving at least a 75% reduction in headache frequency. All estimates were obtained from the included source review and were based on single-arm observational cohorts without concurrent control groups. Negative mean differences indicate improvement from baseline. These findings are presented as contextual evidence on adjunctive pharmacotherapy rather than as evidence for an independent procedural intervention. They should not be interpreted as demonstrating superiority over either component monotherapy.
